# Associations of plant-based foods, red and processed meat, and dairy with gut microbiome in Finnish adults

**DOI:** 10.1007/s00394-024-03406-x

**Published:** 2024-05-16

**Authors:** Mirkka Maukonen, Kari K Koponen, Aki S Havulinna, Niina E Kaartinen, Teemu Niiranen, Guillaume Méric, Anne-Maria Pajari, Rob Knight, Veikko Salomaa, Satu Männistö

**Affiliations:** 1 Finnish Institute for Health and Welfare (THL), Helsinki, Finland; 2 Institute for Molecular Medicine Finland, FIMM-HiLIFE, Helsinki, Finland; 3 Baker Heart and Diabetes Institute, Melbourne, Australia; 4 University of Melbourne, Melbourne, Australia; 5 University of Helsinki, Helsinki, Finland; 6 University of California San Diego, La Jolla, CA USA; 7 Monash University, Melbourne, Australia; 8 La Trobe University, Melbourne, Australia

**Keywords:** Dairy, Diet, Gut microbiome, Plant-based foods, Meat, Sustainability

## Abstract

**Purpose:**

Population-based studies on the associations of plant-based foods, red meat or dairy with gut microbiome are scarce. We examined whether the consumption of plant-based foods (vegetables, potatoes, fruits, cereals), red and processed meat (RPM) or dairy (fermented milk, cheese, other dairy products) are related to gut microbiome in Finnish adults.

**Methods:**

We utilized data from the National FINRISK/FINDIET 2002 Study (*n* = 1273, aged 25–64 years, 55% women). Diet was assessed with 48-hour dietary recalls. Gut microbiome was analyzed using shallow shotgun sequencing. We applied multivariate analyses with linear models and permutational ANOVAs adjusted for relevant confounders.

**Results:**

Fruit consumption was positively (beta = 0.03, SE = 0.01, *P* = 0.04), while a dairy subgroup including milk, cream and ice-creams was inversely associated (beta=-0.03, SE 0.01, *P* = 0.02) with intra-individual gut microbiome diversity (alpha-diversity). Plant-based foods (R^2^ = 0.001, *P* = 0.03) and dairy (R^2^ = 0.002, *P* = 0.01) but not RPM (R^2^ = 0.001, *P* = 0.38) contributed to the compositional differences in gut microbiome (beta-diversity). Plant-based foods were associated with several butyrate producers/cellulolytic species including *Roseburia hominis*. RPM associations included an inverse association with *R. hominis.* Dairy was positively associated with several lactic producing/probiotic species including *Lactobacillus delbrueckii* and potentially opportunistic pathogens including *Citrobacter freundii.* Dairy, fermented milk, vegetables, and cereals were associated with specific microbial functions.

**Conclusion:**

Our results suggest a potential association between plant-based foods and dairy or their subgroups with microbial diversity measures. Furthermore, our findings indicated that all the food groups were associated with distinct overall microbial community compositions. Plant-based food consumption particularly was associated with a larger number of putative beneficial species.

**Supplementary Information:**

The online version contains supplementary material available at 10.1007/s00394-024-03406-x.

## Introduction

The current way we produce and consume food threatens both human health and environmental sustainability. In 2019, the EAT-Lancet Commission launched the planetary health diet, a global reference diet with focus on healthy diet produced in a sustainable way [1]. The main objective of this diet is to increase the consumption of plant-based foods including vegetables, fruits, legumes, whole grains, and nuts while reducing the consumption of animal-sourced foods such as red and processed meat and dairy products.

Another rapidly expanding area of research is understanding the complex relationships between diet, gut microbiome, and human health. Human gut microbiota refers to a complex community of trillions of different micro-organisms residing in the human gut [2]. Diet is considered one of the most important factors influencing composition and function of the gut microbiome, and thus, determining its metabolic outputs that may play a role in human health and disease [3]. Consequently, many diet-associated conditions have been associated with the gut microbiome including obesity [4] and several chronic diseases such as type 2 diabetes [5] and cardiovascular diseases [6]. Thus, it is important to examine the specific roles of different food groups on the gut microbiome.

The associations of plant-based foods, red and processed meat and dairy consumption with gut microbiome have not been extensively examined. Controlled small-scale human trials conducted mainly on individuals with obesity have demonstrated shifts in microbiome diversity [7] or composition [7, 8] and adverse changes in microbial metabolites [7–10] on diets high in animal-based foods and low in carbohydrates during 5-days to 8-weeks. Larger-scale observational studies on healthy French adults [11] and on Chinese middle-aged and elderly [12] have reported inconsistent results on the associations between individual plant-based foods, red meat or dairy with gut microbiome. These studies, however, lacked data on actual consumption of the foods due to the utilized dietary assessment method (a frequency-based food propensity questionnaire (FPQ)) and on some foods in the core of this current study such as dairy subgroups (e.g., fermented milk). These apply also to our previous study, consisting partly of the same study population, where we also used frequency-based FPQ to examine diet quality-microbiome links [13]. Furthermore, another Chinese study on middle-aged and elderly individuals utilized a more detailed dietary assessment method (a semi-quantitative food frequency questionnaire (FFQ)) but focused on associations of vegetables or fruits with gut microbiome [14]. To address these limitations, we used dietary recalls which capture wider range of foods and provide detailed dietary data on the quantitative consumption of these foods allowing for a more comprehensive assessment of the associations of plant-based foods, red meat, or dairy with gut microbiome. Furthermore, in contrast to our previous study where we examined genus-level microbiome associations [13] we now examined species-level associations.

The specific aims of the current study were to examine whether the consumption of plant-based foods (vegetables, potatoes, fruits, cereals), red and processed meat or dairy (fermented milk, cheese, other dairy products) is related to individual gut microbiome diversity (alpha-diversity), inter-individual differences in gut microbiome composition (beta-diversity), and differences in relative abundances of bacterial species in Finnish adults. We also examined how the functional properties of the microbiome relate to these food groups.

## Methods

### Study population

We used data from the National FINDIET 2002 Study [15], a sub-study of the National FINRISK 2002 Study [16]. The FINRISK Studies have been conducted by the Finnish Institute for Health and Welfare (THL) every five years from 1972 until 2012 to monitor risk factors for non-communicable diseases in Finnish adults [16]. FINRISK 2002 comprised of a self-administered health questionnaire and a health examination, involving a random sample from six large geographical areas in Finland drawn from the national population information system (*n* = 8799) (Supplemental Fig. 1). Stool shallow shotgun sequencing was successfully performed for a total of 7231 participants of which additional 20 participants were excluded due low (< 50,000) read counts (*n* = 7211). One third (*n* = 3182, aged 25–64) of the FINRISK participants belonged to the FINDIET 2002 subsample where dietary habits of the participants were assessed by a 48-hour dietary recall (including yesterday and the day before that) [15]. Of those invited, 2045 (64%) completed the recall and 2007 (63%) of the recalls were accepted. After excluding pregnant women (*n* = 10) and those who had a registered purchase of antibacterial medications for systemic use (Anatomical Therapeutic Chemical classification (ATC) code: J01) within six months prior to the baseline examination (*n* = 294), the final data included 1273 participants with available stool samples and dietary recalls.

### Dietary assessment

Food consumption was assessed with a 48-hour dietary recall [15]. Dietary recalls were conducted during the health examination by trained nutritionists who interviewed participants and recorded all foods and beverages consumed. Portion sizes were estimated using commonly used food packaging, household measures and a validated portion size picture booklet [17, 18]. The mean daily energy intake and consumption of food groups (g/day) were assessed using the in-house calculation software Finessi and the Finnish national food composition database (Fineli®) maintained by the THL [19]. Food consumption was calculated at the ingredient level by decomposing mixed dishes into individual ingredients using standard recipes. The main food groups and their subgroups used in the study are presented in Table 1. As we were unable to analyze nuts and seeds separately due to their very low consumption, we included them within the vegetables subgroup and for the same reason we also kept legumes within the vegetables. Similarly due its low consumption, we included ice cream within the other dairy products subgroup. Food variables were used mainly as continuous variables and were z-score transformed for the analysis. The food variables were categorized based on the study specific consumption quartiles for principal coordinates analysis (PCoA) and distance-based redundancy analysis (dbRDA).

**Table 1:** The main food groups and their sub-groups used in the study

Main food group	Sub-groups
Plant-based foods	
	**Vegetables** (including legumes, onions, mushrooms, cabbage, roots, vegetable fruits and leaf vegetables along with nuts and seeds)^1^ **Potatoes** (excluding French fries and potato chips)
	**Fruits** (including citrus, apple and other fruits and berries)
	**Cereals** (rye, oat, barley and wheat)
Red and processed meat	
	**Red meat** (beef, pork, lamb and game)
	**Processed meat** (including sausages and cold cuts)
Dairy	
	**Fermented milk** (including yoghurt, buttermilk, curdled milk, quark)
	**Cheese** (soft, semi-soft, hard)
	**Other dairy products** (milk, cream, ice cream)

^1^ Nuts, seeds and legumes were included in vegetables because of their low consumption levels

### Stool samples

All who participated in the health examination of FINRISK 2002 were asked to donate a stool sample. Those willing were given a stool sampling kit and instructions during the health examination to promptly gather the sample at home at their earliest convenience. Participants collected the samples into 50 ml Falcon tubes without a stabilizing solution and then sent them overnight under Finnish winter conditions to the study personnel preferably on Monday, Tuesday, Wednesday, or Thursday, to ensure optimal preservation of the sample. The samples were immediately stored at − 20 °C and were kept unthawed until sequencing in 2017. The samples were sequenced based on whole-genome, untargeted shallow shotgun sequencing at the University of California San Diego [20]. Normalizing of the samples to 5-ng inputs were done using an Echo 550 acoustic liquid handling robot and the samples were sequenced using Illumina Hi-Seq 4000 (Illumina Inc., San Diego, CA, USA) for paired-end 150-bp reads. The average read count was approximately 900,000 reads per sample. A more detailed description of protocols for DNA extraction and library preparation can be found elsewhere [21]. Quality trimming of the sequences and removal of sequencing adapters was performed using Atropos [22]. After removing human DNA reads by mapping them against the reference genome assembly GRCh38 using Bowtie2 [23], the raw sequences were taxonomically annotated using SHallow shOtGUN profiler (SHOGUN) v1.0.5 [24] by comparing them against complete archaeal, bacterial, and viral genomes in NCBI Reference Sequence Database (NCBI RefSeq) v82 (National Center for Biotechnology Information (NCBI), U.S. National Library of Medicine, Bethesda, MD, USA; May 8, 2017). The classified microbial data were used in a compositional form, meaning their relative abundances were calculated by scaling their raw counts to the total sum of reads. For taxa analyses, the data were filtered to bacterial taxa and down to a core microbiome including any bacterial species with a minimum abundance of 0.01% and a prevalence of at least 1% across all samples, similar to Salosensaari et al. (2021) [21].

### Anthropometric, sociodemographic, and lifestyle variables

Trained nurses at the study site measured weight and height using standardized international protocols with participants wearing light clothing and no shoes [25]. Height was measured to the nearest 0.1 cm using a wall-attached stadiometer and weight to the nearest 0.1 kg using a beam balance scale. BMI was calculated as kg/m². Participants’ age was calculated based on the birth date and study date, and sex was self-reported. The self-administered questionnaire included questions on participant´s smoking history and current smoking habits. For the analysis two groups were formed: current smokers and nonsmokers who had not smoked in the last 6 months. Information on medicines which could potentially affect the microbiome (metformin, ATC code A10BA02, psycholeptics, ATC code NO5, and psychoanaleptics, ATC code NO6) in addition to the excluded systemic antimicrobial medicines (ATC code JO1) was acquired from the prescription medicine purchase register maintained by the Social Insurance Institution of Finland. Participants were linked to the register through the unique personal identifier assigned to each Finnish citizen. In contrast to assessing the use of systemic antimicrobial medication, an individual was flagged as using these other drugs if he/she had at least 3 separate purchase events including a purchase within 4 months prior to baseline investigation.

### Statistical analyses

The analyses were conducted jointly for men and women because the results in general were similar by sex. Characteristics of the study participants are reported as means with their standard deviations (SD) for continuous variables and as percentages for categorical variables.

Alpha-diversity refers to intra-individual diversity of the microbiome and it was measured using the Shannon index [26]. The associations between alpha-diversity and the main food groups and their subgroups were assessed using linear regression analysis. Beta-diversity refers to inter-individual diversity of the gut microbiome and thus acts as a measure of compositional difference. It was measured using the Bray-Curtis dissimilarity score [27]. Permutational multivariate analysis of variance (PERMANOVA) [28] was used to assess the amount of compositional variation in microbiomes between individuals were explained by main food groups and their subgroups. Principal coordinates analysis (PCoA) was used to assess and visualize clustering of microbiomes in the highest and the lowest consumption quartile of each main food group. PCoA was paired with the function “factorfit” from the *vegan* package (version 2.6-4) to test whether the averages of the PCoA ordination scores of the highest and the lowest consumption quartiles of the main food groups differ significantly (= clustering of the microbiomes). Distance-based redundancy analysis (dbRDA) [29] is an extension of PCoA to model multivariate data and it was used to assess the amount the main food groups and confounding factors together explain of the compositional variation of gut microbiomes between individuals (= constrained variance) and visualizing the direction of the associations. PERMANOVA, factorfit, and dbRDA were run with 999 permutations.

In per-taxa analyses we used a multivariate analysis by linear models (*MaAsLin2*, version 1.16.0) [30] to analyze associations of each main food group and their subgroups with all taxa at species level. Benjamini-Hochberg false discovery rate (FDR) corrected *P* values were used to adjust for multiple comparisons. Prior to analysis the relative abundances of the taxa were centered log-ratio (CLR) –transformed. For the cluster analyses, the taxa with significant associations with the main food groups from per-taxa analysis were clustered based on proportionality using Ward minimum variance method and the optimal number of clusters was determined using Kelley-Gardner-Sutcliffe penalty function [31]. The results were visualized with a heatmap.

A pathway analysis was conducted between Kyoto Encyclopedia of Genes and Genomes orthology (KO) groups and the food groups using linear regression analysis. The relative abundances of KO-groups for each sample were obtained from the strain-level outputs of SHOGUN and the data on KO-groups were log10-transformed prior to analysis. Benjamini-Hochberg FDR corrected *P* values were used to adjust for multiple comparisons. Statistically significant associations of main food groups were further visualized using *FuncTree 2* [32].

Analyses were controlled for potential confounding factors based on prior literature. These included age, sex, BMI, smoking status, usage of possible microbiome-altering medications (metformin and psycholeptics/analeptics) and total energy intake. The level of statistical significance for analyses was set at a *P* value < 0.05 (also for the FDR corrected *P* values). Statistical analyses were performed using R version 3.6.3 [33] and the following packages *phyloseq*, *microbiome*, *vegan*, *maptree* and *ComplexHeatmap* [31, 34–37].

## Results

### Selected characteristics of the study sample

Of the participants, 46% were men and the mean age was 47 years (Table 2). In all, 25% were current smokers and the mean BMI was 26.8 kg/m². Potentially microbiome altering medicines were used by 7% of the participants of which 1% used metformin and 6% psycholeptics/analeptics. The overall mean energy intake was about 7900 kJ/day, and the mean consumption of plant-based foods was 498 g/day (Q1: 269 g/day, Q4: 768 g/day), red and processed meat 98 g/day (Q1:19 g/day, Q4:204 g/day) and dairy 436 g/day (Q1:123 g/day, Q4: 851 g/day).

**Table 2:** Characteristics of the study sample as means (standard deviation (SD)) or percentages

			Plant-based food consumption quartiles			Red and processed meat consumption quartiles			Dairy consumption quartiles	
	All		Q1 (lowest)	Q4 (highest)		Q1 (lowest)	Q4 (highest)		Q1 (lowest)	Q4 (highest)
	*n* = 1273		*n* = 319	*n* = 318		*n* = 319	*n* = 318		*n* = 319	*n* = 318
Sex, male, %	45.5		41.4	47.2		26.0	78.0		43.6	59.4
Age, years	46.8 (11.2)		44.1 (11.6)	48.6 (10.8)		47.7 (11.5)	46.4 (11.3)		47.5 (10.1)	45.7 (12.3)
Current smoker, %	24.8		34.2	17.0		17.2	31.4		22.3	25.5
BMI, kg/m²	26.8 (4.6)		27.0 (4.6)	26.6 (4.5)		26.3 (4.6)	27.2 (4.7)		26.8 (4.6)	27.0 (4.9)
Medicine use, %	7.1		6.2	6.9		6.9	6.3		7.2	7.9
Psycholeptic/analeptic user, %	6.2		6.0	6.0		6.0	5.3		6.3	6.9
Metfromin user, %	0.8		0.6	0.9		1.3	0.9		0.9	1.6
Energy, kJ/day	7892 (2645)		6839 (2349)	9112 (2848)		6573 (1940)	9944 (2718)		7096 (2533)	9427 (2762)
Carbohydrate, E%	48.8 (7.5)		47.1 (8.4)	50.9 (6.8)		51.4 (7.6)	45.6 (6.8)		48.9 (8.5)	48.3 (7.4)
Fiber, g/day	20.7 (9.3)		13.7 (5.5)	28.9 (10.7)		19.8 (8.5)	22.8 (10.8)		19.8 (10.5)	23.3 (9.7)
Protein, E%	16.2 (3.4)		16.3 (3.7)	16.0 (3.2)		15.7 (3.6)	16.6 (3.6)		15.0 (3.2)	17.4 (3.6)
Fat, E%	32.9 (7.4)		34.9 (7.5)	30.5 (7.1)		30.4 (7.1)	35.9 (6.7)		33.8 (8.0)	32.3 (7.8)
SAFA, E%	13.4 (3.9)		14.5 (4.0)	11.9 (3.6)		12.4 (4.1)	14.3 (3.6)		12.9 (4.1)	13.6 (4.2)
Plant-based foods, g/day	497.5 (212.1)		268.6 (60.5)	768.0 (148.3)		500.9 (202.4)	518.5 (214.5)		491.4 (221.4)	525.4 (202.1)
Vegetables, g/day	133.7 (96.9)		75.5 (52.5)	212.5 (118.3)		153.1 (106.6)	124.5 (90.7)		147.1 (111.9)	121.9 (88.6)
Potatoes, g/day	90.8 (75.3)		51.8 (51.1)	125.8 (87.9)		67.4 (61.4)	121.4 (86.9)		83.5 (73.9)	108.1 (82.5)
Fruits, g/day	138.1 (125.3)		42.3 (50.4)	258.6 (143.8)		161.2 (133.7)	114.123.2)		131.7 (126.5)	139.0 (128.8)
Cereals, g/day	135.0 (70.1)		99.0 (46.1)	171.1 (89.9)		119.3 (63.8)	158.0 (76.6)		129.1 (80.0)	156.4 (73.5)
Red and processed meat, g/day	97.6 (78.2)		96.5 (77.9)	104.7 (83.4)		18.6 (13.6)	204.2 (69.5)		96.58 (86.3)	112.4 (84.4)
Red meat, g/day	54.6 (55.9)		50.6 (52.7)	62.0 (59.3)		10.1 (12.4)	110.8 (70.7)		54.6 (58.5)	60.5 (61.1)
Processed meat, g/day	43.0 (53.7)		45.9 (59.8)	42.7 (52.7)		8.5 (11.3)	93.4 (75.1)		42.0 (58.4)	51.9 (59.6)
Dairy, g/day	436.4 (302.2)		396.5 (289.0)	461.2 (327.7)		367.3 (232.5)	491.4 (332.6)		122.69 (59.6)	851.4 (253.4)
Fermented milk, g/day	107.2 (145.0)		86.5 (116.2)	125.4 (153.6)		113.4 (135.5)	98.4 (144.6)		23.1 (45.6)	183.2 (202.3)
Cheese, g/day	38.3 (37.0)		35.1 (35.5)	40.3 (42.1)		38.9 (32.4)	41.5 (43.7)		31.9 (31.5)	43.5 (41.8)
Other dairy products, g/day	290.9 (279.3)		275.0 (274.9)	296.0 (298.9)		218.1 (211.0)	351.5 (311.3)		67.7 (55.9)	624.7 (313.6)
SAFA; saturated fatty acids										

### Individual microbial diversity (alpha-diversity)

None of the main food groups were significantly associated with intra-individual variation in microbiome diversity (Shannon alpha-diversity index, *P* > 0.05; Supplemental Table 1). Of the subgroups, fruit consumption was positively (beta = 0.03, SE = 0.01, *P* = 0.04) and other dairy products inversely (beta=-0.03, SE 0.01, *P* = 0.021) associated with alpha-diversity, whereas no significant associations occurred with the other subgroups (*P* > 0.05).

### Compositional differences in gut microbiome (beta-diversity)

All the main food groups, except red and processed meat (R^2^ = 0.001, *P* = 0.38), explained small but significant amounts of the compositional variation between individuals’ microbiomes (plant-based foods: R^2^ = 0.001, *P* = 0.03, dairy: R^2^ = 0.002, *P* = 0.01) (Supplemental Table 2). All the subgroups of plant-based foods (except for potatoes, *P* = 0.48) and dairy (except for fermented milk, *P* = 0.27) were also associated with compositional differences in microbiome (*P* < 0.05), whereas no associations were found when red meat and processed meat were analyzed separately (*P* > 0.05).

**Fig. 1:**
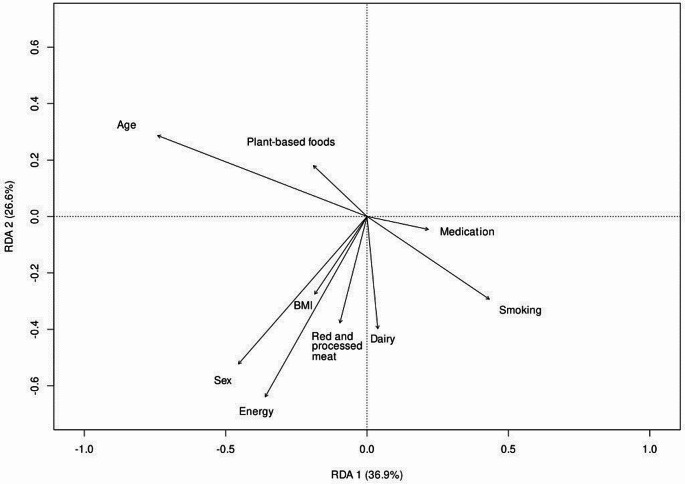
The directions of the associations of the main food groups and confounding factors with microbiome based on distance-based redundancy analysis (dbRDA) results related to beta-diversity Bray-Curtis dissimilarity scores. The amount of constrained variance (i.e., the percentage of variance explainable by the main food groups and confounding factors) explain of the compositional variation of gut microbiomes between individuals by the first two axes (RDA1 and RDA2) is displayed in parenthesis

The directions of the associations of the main food groups and confounding variables with microbiome were examined by performing a dbRDA. Together these variables (= constrained variation) accounted for 2.71% of the total variance related to gut microbiome (*P* = 0.001). The first two axes together captured most of the constrained variation (63.5%) i.e., the variation explained by the chosen set of explanatory variables and 1.72% of the total variance (Fig. 1). Of the main food groups, plant-based foods together with age were positively correlated with the second axis (explained constrained variance: 26.6%), whereas red and processed meat, dairy and the other confounding factors were inversely associated with the same axis.

Furthermore, we examined the clustering of the gut microbiomes by consumption quartiles of the main food groups by performing a PCoA. No significant clustering of microbiomes was observed between the highest and the lowest consumption quartiles in any of the main food groups (*P* > 0.05) (Supplemental Fig. 2).

### Per-taxa and cluster analysis

**Fig. 2:**
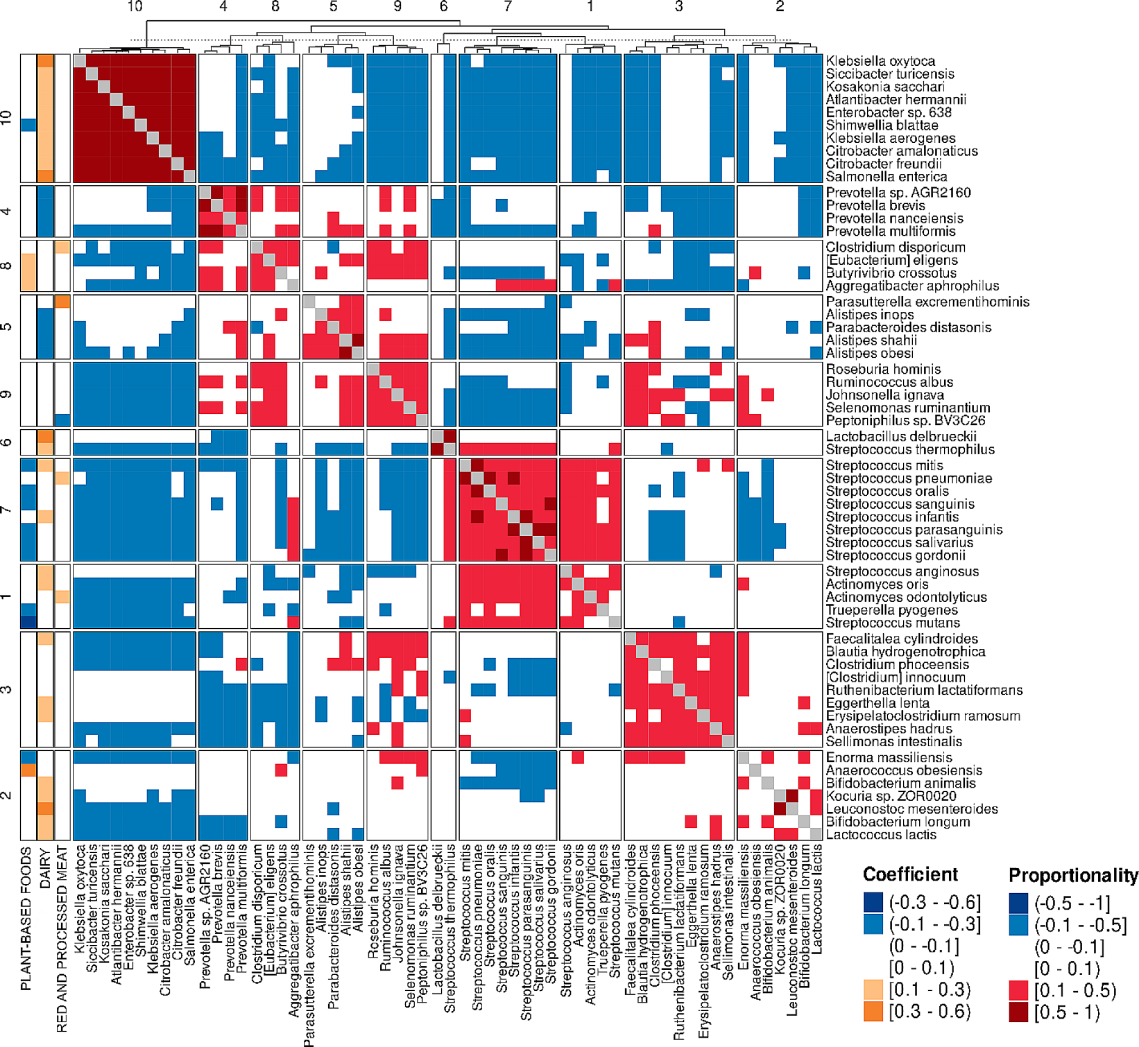
A heatmap of the results from per taxa and cluster analysis. Associations between the main food groups and species level taxa were determined with multivariate association with linear models (MaAsLin) tool. The taxa with significant associations with the main food groups were then clustered based on proportionality. To adjust for multiple comparisons Benjamini-Hochberg FDR corrected *P* values were used. The models were adjusted for age, sex, BMI, smoking status, usage of possible microbiome-altering medications (metformin and psycholeptics/analeptics) and total energy intake

We analyzed individual taxa associations of each main food group and their subgroups and further conducted a cluster analysis for the taxa with significant associations with the main food groups (Fig. 2, Supplemental Table 3). Plant-based foods were associated with 21 individual bacterial species of which 14 were inverse and seven positive associations. Several inversely associated species were from genus *Streptococcus*, whereas most of the positively associated species were from family *Lachnospiraceae* representing several genera and species including *Roseburia hominis* and *Eubacterium eligens*. Inverse associations with several species from genus *Streptococcus* were also seen within each subgroup of plant-based foods (except for potatoes), whereas positive associations with genera from family *Lachnospiraceae* were mainly accounted for by fruits (Supplemental Table 4). Cereals and vegetables were positively associated with species from genus *Prevotella*, whereas cereals were positively, and vegetables inversely associated with several species from genus *Bifidobacterium*. Red and processed meats were associated with seven species of which four were positive and three inverse associations. Positively associated species were from genera *Parasutterella, Streptococcus, Actinomyces* and *Clostridium*. Two of the inversely associated species were from family *Lachnospiraceae* including *Roseburia hominis.* Inverse association with *Roseburia hominis* was accounted for by processed meat subgroup, whereas red meat subgroup associations included inverse associations with species from genus *Parasutterella* and two species from genus *Prevotella* (Supplemental Table 5). Dairy was associated with 36 species of which 27 were positively and nine inversely associated. Among positive associations were several lactic acid producing species and species from family *Enterobacteriaceae*. Inversely associated species were mostly from genus *Prevotella* and family *Rikenellaceae*. Positive lactic acid producing species associations of dairy were mainly accounted for by fermented milk subgroup and cheese, whereas inverse association with genus *Prevotella* was observed with fermented milk and other dairy products and positive associations with family *Enterobacteriaceae* were seen also with other dairy products (Supplemental Table 6).

The cluster analysis revealed 10 clusters of which plant-based foods consumption was positively associated with two (Cluster 8; *P* < 0.001, Cluster 9; *P* = 0.001) and inversely with two clusters (Cluster 1; *P* < 0.001, Cluster 7; *P* = 0.001) (Fig. 2, Supplemental Table 3). Red and processed meat consumption was positively associated with two clusters (Cluster 1; *P* = 0.001, Cluster 7; *P* = 0.031) and inversely with one (Cluster 9; *P* = 0.001). Dairy consumption was positively associated with six clusters (Cluster 1; *P* = 0.008, Cluster 2; *P* = 0.003, Cluster 3; *P* = 0.016, Cluster 6; *P* < 0.001, Cluster 7; *P* = 0.003, Cluster 10; *P* < 0.001) and inversely with two (Cluster 4; *P* = 0.001, Cluster 5; *P* < 0.001).

### Pathway analysis

**Fig. 3:**
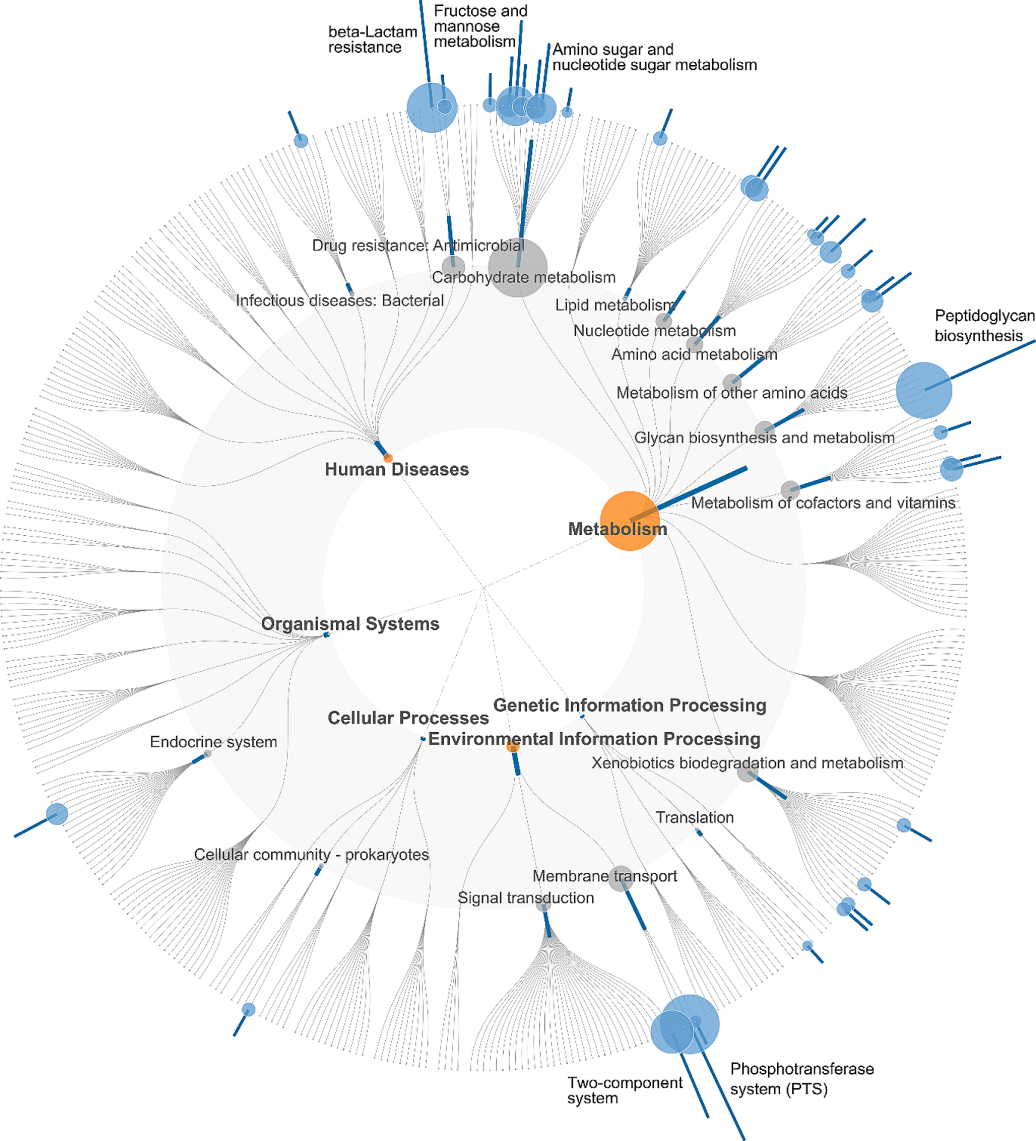
Functional microbial pathways positively associated with dairy consumption illustrated with a FuncTree 2 plot. Each layer of the tree corresponds to functional category starting from the center: KEGG (level 1), KEGG (level 2), and the outermost layer corresponds to KEGG Pathway. Each layer contains nodes that correspond to the biological functions assignable to that functional category. Node sizes reflect the sum of statistically significantly (Benjamini-Hochberg FDR corrected *P* value < 0.05) KO groups associated to that node. *P* values were determined with linear regression models adjusted for age, sex, BMI, smoking status, usage of possible microbiome-altering medications (metformin and psycholeptics/analeptics) and energy intake. KEGG, Kyoto Encyclopedia of Genes and Genomes

**Fig. 4:**
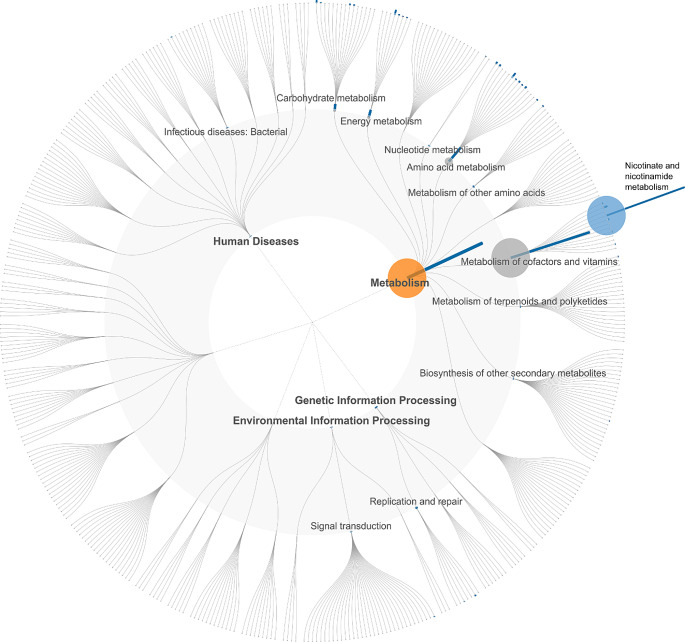
Functional microbial pathways inversely associated with dairy consumption illustrated with a FuncTree 2 plot. Each layer of the tree corresponds to functional category starting from the center: KEGG (level 1), KEGG (level 2), and the outermost layer corresponds to KEGG Pathway. Each layer contains nodes that correspond to the biological functions assignable to that functional category. Node sizes reflect the sum of statistically significantly (Benjamini-Hochberg FDR corrected *P* value < 0.05) KO groups associated to that node. *P* values were determined with linear regression models adjusted for age, sex, BMI, smoking status, usage of possible microbiome-altering medications (metformin and psycholeptics/analeptics) and total energy intake. KEGG, Kyoto Encyclopedia of Genes and Genomes

Of the main food groups, no significant associations were found between plant-based foods or red and processed meat with KO groups (Benjamini-Hochberg FDR *P* > 0.05) (data not shown). Dairy consumption was associated with 75 KO groups from the total 3017 analyzed groups. Of these 41 were positive and 34 inverse associations (Figs. 3 and 4, Supplemental Table 7). The positive associations of dairy consumption were found in all functional groups (KEGG Level 1 annotations). In particular, several pathways in the functional group of metabolism were enriched, including peptidoglycan biosynthesis related to glycan biosynthesis and metabolism and carbohydrate metabolism related fructose and mannose metabolism and amino sugar and nucleotide sugar metabolism (Fig. 3). Prominent associations were also observed in the functional group of environmental information processing and more specifically signal transduction (two-component system) and membrane transport (phosphotransferase system) and further an association signal in the pathway related to beta-Lactam resistance. The most prominent inverse association was related to metabolism of cofactors and vitamins and more specifically nicotinate and nicotinamide metabolism (Fig. 4). Of the food subgroups, fermented milk showed positive associations with 88 KO groups of which 25 were shared with the main dairy group, including pathways related to glycan biosynthesis, carbohydrate metabolism, environmental information processing, and beta-lactam resistance (Supplemental Table 7). Other dairy products showed an inverse association with a single KO group (K01950) related to nicotinate and nicotinamide metabolism. Vegetables were associated with 140 KO groups of which 17 were positive associations and 123 were inverse. Positive associations were mainly related to carbohydrate (e.g., amino sugar and nucleotide sugar metabolism) and lipid metabolism. Negative associations with vegetables were observed in all functional groups (KEGG 1 level annotations) including several pathways related to metabolism (e.g., amino acids, energy, lipid, and cofactors and vitamins). Cereals were positively associated with 28 KO groups, mainly related to carbohydrate metabolism, including starch and sucrose metabolism.

## Discussion

In this population-based study of Finnish adults we examined the associations of plant-based foods, red and processed meat, and dairy and their subgroups with gut microbiome. We found that fruit consumption was positively and a subgroup of dairy including milk, cream and ice-cream was inversely associated with intra-individual diversity (alpha-diversity) and that plant-based foods (except for potatoes), dairy and their subgroups (except for fermented milk) were associated with compositional differences between individuals (beta-diversity) in microbiome. Plant-based foods, red meat and dairy were also associated with relative abundances of distinctive bacterial species. Furthermore, dairy and of the subgroups fermented milk, other dairy products, vegetables, and cereals were associated with microbial pathways.

Findings regarding alpha- and beta diversity from previous larger-scale observational studies are inconsistent. A French study on healthy adults (*n* = 862) found that fresh fruit consumption frequencies were positively and meat (not specified) or processed meat were inversely associated with alpha-diversity, whereas fresh/cooked vegetables, cooked fruits, legumes, dairy products, or cheese were not associated with alpha-diversity [11]. Of these fresh and cooked fruits and cheese, however, were associated with beta-diversity. A recent Chinese study on middle-aged and elderly people (*n* = 1879) found that fruit but not vegetable consumption (g/day) was associated with alpha and beta-diversity [14]. Another Chinese study (*n* = 702) also on middle aged and elderly found no association between consumption frequencies of fruits, vegetables, whole or refined grains, red meat or dairy products with alpha-diversity, whereas of these whole grains and vegetables were associated with beta-diversity [12]. In our previous study (*n* = 4930) fruits and berries consumption (frequencies) were positively associated with alpha diversity which is in line with our current study, however differing from this current study were the positive associations found also for fiber-rich breads (representing cereals) and low-fat cheese (representing dairy group) [13]. As for beta-diversity all the aforementioned foods, in addition to vegetables and red-meat products (unlike in the current study), were also associated with beta-diversity.

Geographical variations in microbial compositions across studies with diverse populations and food cultures may contribute to observed differences [38]. Another potential explanation could be related to the differences in utilized dietary assessment methods potentially influencing the findings to some extent.

In species level analyses, consumption of plant-based foods was positively associated with cellulolytic species such as *Ruminococcus albus* [39] and several known short-chain fatty acid (SCFA)-producing species from *Lachnospiraceae* family such as *Eubacterium eligens*, *Roseburia hominis* and *Butyrivibrio crossotus* [40]. Red and processed meat consumption was associated with only few individual bacterial species including a positive association with *Clostridium disporicum*, previously linked to secondary bile acid production [41] and an inverse association with *R. hominis* (accounted for by processed meat subgroup). Also our previous study [13] as well as the other aforementioned observational studies [11, 12, 14] have linked *Ruminococcus, Roseburia* or *Eubacterium* genera to higher consumption of several plant-based foods including fruits [11, 13, 14], vegetables [12, 13] or fiber-rich breads [13]. In the current study these associations were also seen in all subgroups of the plant-based foods (except for potatoes) in general. Furthermore, a US trial (*n* = 9) examining short-term effects of plant-based diet and animal-based diets on gut microbiome found that species from *Ruminococcus, Roseburia* and *Eubacterium* genera were more abundant with plant-based diet [7]. The SCFAs, mainly butyrate, acetate, and propionate, can affect human health including potential benefits for insulin sensitivity and management of type 2 diabetes and obesity [42]. Reduced fecal SCFA levels have also been associated with a higher risk of colorectal cancer [43]. Furthermore, we also found that the plant-based subgroups cereals and vegetables were associated with species from the *Prevotella* genus, which is in line with previous literature showing an association with plant rich diets low in animal-sourced foods [44], for example a US study on healthy participants (*n* = 98) showed a positive association of *Prevotella* genus with diets rich in plant-derived fibers and carbohydrates and inverse association with fatty and amino acid rich diets [45]. Cereals were also positively associated with several species from genus *Bifidobacterium*. This was also seen in a recent U.S. study on healthy adults (*n* = 343) [46] and in our previous study with fiber rich bread [13]. A systematic review on human intervention studies (*n* = 40 studies) for the effects of cereal fibers on gut microbiota composition in healthy adults indicated increases in *Bifidobacterium* species abundances by wheat, oat and barley but not with rye [47]. Furthermore, we found that dairy consumption was positively associated (mainly accounted for by fermented dairy subgroup) with several lactic acid bacteria including commonly used starter cultures for fermented dairy products such as *Lactobacillus delbrueckii* and *Streptococcus thermophilus* [48]. Inverse associations were found with several species of *Prevotella* genus. These findings are in line with a recent systematic review of randomized controlled trials (*n* = 468) on effects of dairy consumption on human gut microbiome which concluded that dairy consumption increased the abundance of the beneficial genera *Lactobacillus* and *Bifidobacterium* and there were also indications of reductions in *Prevotella* species [49]. Furthermore, we found that dairy consumption was positively associated with several species from Enterobacteriaceae family (mainly accounted for by other dairy products subgroup) such as *Citrobacter freundii* and *Klebsiella oxytoca.* Although these species have been recognized as natural species of microbiome related to dairy and dairy products, they may also be potentially opportunistic pathogens to humans [50–52].

Our pathway analysis indicated that of the main food groups dairy consumption may be associated with several microbial pathways including those related to carbohydrate metabolism and glycan biosynthesis and metabolism. Of the subgroups, similar pathway associations were observed with fermented milk and for the vegetables and cereals several associations were also detected in pathways related to metabolism including carbohydrate or lipid metabolism. Further studies are, however, needed to explore the importance of these findings and information is also needed on the possible causal mechanisms behind these associations.

The strength of our study included a population-based approach using a representative random population sample. We were also able to consider several crucial factors influencing the gut microbiome, however, there are certain factors we were unable to account for including the use of other medications (e.g., laxatives, proton pump inhibitors), recent gastrointestinal surgery or recent substantial diet alterations. Furthermore, whole metagenomic shallow shotgun sequencing utilizing SHOGUN optimized to be used with this kind of sequencing provides more robust taxonomic and functional information compared to 16 S RNA amplicon sequencing [20]. However, while superior to 16 S RNA amplicon sequencing, shallow shotgun sequencing is less accurate than deep sequencing in capturing genetic features, requiring some caution in interpreting functional results [53]. Furthermore, the cross-sectional design of this study cannot reveal causality. Also, dietary intake assessment relied on self-reported data, possibly affected by memory and reporting biases. The 48-hour dietary recalls covered two consecutive days, and there was likely some correlation between the days. However, dietary recalls provide more detailed insights into food types and quantities, compared to questionnaires and may be valuable in capturing temporal associations with the gut microbiome. This is of interest since, despite the perceived stability of the gut microbiome, rapid shifts can occur due to diet [7, 54]. The temporal gap between the stool collections and dietary recalls in our study was relatively brief, ranging from a matter of day(s) to at most a few weeks. As a limitation, dietary recalls may not be an accurate estimate of long-term dietary habits. It’s noteworthy, however, that the examined food groups are prevalent foods in Finnish diets, which increases the likelihood of detecting associations and strengthens the reliability of our findings with a potential to reflect even more enduring dietary patterns. Additionally, in FINDIET 2002, the 48-hour dietary recalls were compared with a 5-day dietary information (48-hour dietary recall and a 3-day food diary) within a subsample, which demonstrated comparable results [15]. A recent study on British adults similarly supported the effectiveness of a 48-hour recall in characterizing dietary patterns when compared to a 5-day food diary [55]. It is also known that health-conscious people are more likely to participate in health surveys, however, our participation rate was rather high (64%) and therefore the bias related to the healthy participant effect is likely to be small [56]. Furthermore, the geographical differences in gut microbiome composition may limit the generalizability of our results to some extent in different sociocultural, and environmental settings, for example [38].

In conclusion, we found that consumption of plant-based foods and dairy contributed to the compositional differences in gut microbiome in terms of beta-diversity and that plant-based foods, red and processed meat and dairy were associated with distinct overall microbial community compositions. Plant-based food consumption particularly was associated with a larger number of putative beneficial species on average. These findings indicate that a shift towards more sustainable and healthier diets with more plant-based foods and moderate dairy consumption could also benefit our gut microbiome.

## Electronic supplementary material

Below is the link to the electronic supplementary material.

Supplementary Material 1

## Data Availability

The dataset used is available upon request through the Findata permit procedure. https://www.findata.fi/en/.
