# Supplementary material for: Associations of plant-based foods, red and processed meat, and dairy with gut microbiome in Finnish adults
Source: Eur J Nutr. 2024 May 16;63(6):2247–60. doi: 10.1007/s00394-024-03406-x (PMC11377619; doi:10.1007/s00394-024-03406-x)
Supplement: Supplementary file 1 — Supplementary Material 1 [file 394_2024_3406_MOESM1_ESM.docx]

**Supplementary Information**

**Title:** Associations of plant-based foods, red and processed meat, and dairy with gut microbiome in Finnish adults

**Journal**: European Journal of Nutrition

**Authors**: Mirkka Maukonen^1^, Kari K Koponen^1^, Aki S Havulinna^1,2^, Niina E Kaartinen^1^, Teemu Niiranen^1^, Guillaume Méric^3,4,7,8^, Anne-Maria Pajari^5^, Rob Knight^6^, Veikko Salomaa^1^, Satu Männistö^1^

^1^ Finnish Institute for Health and Welfare (THL), Helsinki, Finland

^2^ Institute for Molecular Medicine Finland, FIMM-HiLIFE, Helsinki, Finland.

^3^ Baker Heart and Diabetes Institute, Australia

^4^ University of Melbourne, Australia

^5^ University of Helsinki, Helsinki, Finland

^6^ University of California San Diego, La Jolla, CA, USA

⁷ Monash University, Melbourne, Australia

⁸ La Trobe University, Melbourne, Australia

**Corresponding author**: Mirkka Maukonen, [mirkka.maukonen@thl.fi](mailto:mirkka.maukonen@thl.fi)

Supplemental Fig 1-2

Supplemental Tables 1-7

**SUPPLEMENTAL FIG 1.** Flowchart of the study sample.

**SUPPLEMENTAL FIG 2.** Clustering of the gut microbiomes of the individuals in the highest and lowest quartiles of the main food group consumptions based on the principal coordinate analysis (PCoA) of Bray-Curtis dissimilarity scores on the first 2 principal coordinates. *P* values to test whether the averages of the PCoA ordination scores of the highest and the lowest consumption quartiles of the main food groups differed significantly was determined with a factorfit function from the Vegan package.

| **Supplemental Table 1.** Associations between food groups and Shannon α-diversity index using multivariable linear regression analysis. | | | |
| --- | --- | --- | --- |
|  | **β** | **SE** | ***P*** |
| **Plant-based foods** | 0.007 | 0.013 | 0.61 |
| Vegetables¹, g/day | -0.007 | 0.012 | 0.58 |
| Potatoes, g/day | -0.015 | 0.013 | 0.26 |
| Fruits², g/day | 0.025 | 0.012 | 0.042 |
| Cereals³, g/day | -0.006 | 0.015 | 0.71 |
|  |  |  |  |
| **Red and processed meat** | -0.021 | 0.014 | 0.13 |
| Red meat⁴, g/day | -0.014 | 0.013 | 0.27 |
| Processed meat⁵, g/day | -0.011 | 0.013 | 0.40 |
|  |  |  |  |
| **Dairy** | -0.017 | 0.013 | 0.20 |
| Fermented milk⁶, g/day | 0.016 | 0.012 | 0.18 |
| Cheese, g/day | 0.018 | 0.013 | 0.15 |
| Other dairy products⁷, g/day | -0.030 | 0.013 | 0.021 |
| SE; standard error |  |  |  |
| Adjusted for age, sex, BMI, smoking, potentially microbiome altering medication and total energy intake (kJ/day).  ¹ All vegetables including legumes along with nuts, seeds, excluding potatoes.  ² All fruits and berries.  ³ Rye, oat, barley and wheat.  ⁴ Beef, pork, lamb and game  ⁵ Including sausages and cold cuts  ⁶ Including yoghurt, buttermilk, curdled milk  ⁷ Milk, cream, ice cream | | | |

| **Supplemental Table 2.** Associations between food groups and Bray-Curtis beta-diversity index using multiadjusted permutational ANOVAs. | | |
| --- | --- | --- |
|  | **R^2^** | ***P*** |
| **Plant-based foods** | 0.001 | 0.034 |
| Vegetables¹, g/day | 0.002 | 0.004 |
| Potatoes², g/day | 0.001 | 0.48 |
| Fruits³, g/day | 0.002 | 0.011 |
| Cereals⁴, g/day | 0.002 | 0.001 |
|  |  |  |
| **Red and processed meat** | 0.001 | 0.38 |
| Red meat⁵, g/day | 0.001 | 0.13 |
| Processed meat⁶, g/day | 0.001 | 0.63 |
|  |  |  |
| **Dairy** | 0.002 | 0.009 |
| Fermented milk⁷, g/day | 0.001 | 0.27 |
| Cheese, g/day | 0.002 | 0.013 |
| Other dairy products⁸, g/day | 0.001 | 0.029 |
| Adjusted for age, sex, BMI, smoking, potentially microbiome altering medication and total energy intake (kJ/day).  ¹ All vegetables including legumes along with nuts, seeds, excluding potatoes.  ² Excluding French fries and potato chips.  ³ All fruits and berries.  ⁴ Rye, oat, barley and wheat.  ⁵ Beef, pork, lamb and game  ⁶ Including sausages and cold cuts  ⁷ Including yoghurt, buttermilk, curdled milk  ⁸ Milk, cream, ice cream | | |

| **Supplemental Table 3.** Species level taxa and cluster analyses associations of each main food group. | | | | | | | | | |  |  |
| --- | --- | --- | --- | --- | --- | --- | --- | --- | --- | --- | --- |
|  |  | **Plant-based foods** | |  | **Red and processed meat** | |  | **Dairy** | |  |  |
| Phylum/Class/Order /Family /**Genus Species** |  | Beta (SE) | *P*¹ |  | Beta (SE) | *P*¹ |  | Beta (SE) | *P*¹ |  |  |
| **Cluster 1** |  | **-0.15 (0.03)** | **<0.001** |  | **0.10 (0.03)** | **<0.001** |  | **0.07 (0.03)** | **0.008** |  |  |
| *Actinobacteria/Actinomycetia/Actinomycetales/Actinomycetaceae/****Trueperalla pyogenes*** |  | -0.14 (0.05) | 0.044 |  | - | - |  | - | - |  |  |
| *Actinobacteria/Actinomycetia/Actinomycetales/Actinomycetaceae/****Actinomyces odontolyticus*** |  | - | - |  | 0.17 (0.05) | 0.009 |  | - | - |  |  |
| *Actinobacteria/Actinomycetia/Actinomycetales/Actinomycetaceae/****Actinomyces oris*** |  | - | - |  | - | - |  | 0.15 (0.05) | 0.047 |  |  |
| *Firmicutes/Bacilli/Lactobacillales/Streptococcaceae/****Streptococcus mutans*** |  | -0.32 (0.07) | 0.001 |  | - | - |  | - | - |  |  |
| *Firmicutes/Bacilli/Lactobacillales/Streptococcaceae/****Streptococcus anginosus*** |  | - | - |  | - | - |  | 0.24 (0.08) | 0.020 |  |  |
|  |  |  |  |  |  |  |  |  |  | |  |
| **Cluster 2** |  | 0.001 (0.04) | 0.98 |  | -0.04 (0.05) | 0.36 |  | **0.12 (0.04)** | **0.003** | |  |
| *Firmicutes/Tissierellia/Tissierellales/Peptoniphilaceae/****Anaerococcus obesiensis*** |  | 0.33 (0.08) | 0.001 |  | - | - |  | - | - |  |  |
| *Actinobacteria/Actinomycetia/Bifidobacteriales/Bifidobacteriaceae/****Bifidobacterium animalis*** |  | - | - |  | - | - |  | 0.24 (0.07) | 0.004 |  |  |
| *Actinobacteria/Actinomycetia/Micrococcales/Micrococcaceae/****Kocuria sp. ZOR0020*** |  | - | - |  | - | - |  | 0.23 (0.08) | 0.031 |  |  |
| *Firmicutes/Bacilli/Lactobacillales/Lactobacillaceae/****Leuconostoc mesenteroides*** |  | - | - |  | - | - |  | 0.52 (0.09) | <0.001 |  |  |
| *Actinobacteria/Actinomycetia/Bifidobacteriales/Bifidobacteriaceae/****Bifidobacterium longum*** |  | - | - |  | - | - |  | 0.15 (0.05) | 0.030 |  |  |
| *Firmicutes/Bacilli/Lactobacillales/Streptococcaceae/****Lactococcus lactis*** |  | - | - |  | - | - |  | 0.27 (0.09) | 0.024 |  |  |
| *Actinobacteria/Coriobacteriia/Coriobacteriales/Coriobacteriaceae/****Enorma massiliensis*** |  | -0.12 (0.04) | 0.023 |  | - | - |  | - | - |  |  |
|  |  |  |  |  |  |  |  |  |  |  |  |
| **Cluster 3** |  | -0.03 (0.02) | 0.24 |  | -0.04 (0.03) | 0.17 |  | **0.06 (0.02)** | **0.016** |  |  |
| *Firmicutes/Erysipelotrichia/Erysipelotrichales/Erysipelotrichaceae/****[Clostridium] innocuum*** |  | -0.08 (0.03) | 0.030 |  | - | - |  | - | - |  |  |
| *Firmicutes/Clostridia/Eubacteriales/Oscillospiraceae/****Ruthenibacterium lactatiformans*** |  | - | - |  | - | - |  | 0.10 (0.03) | 0.012 |  |  |
| *Firmicutes/Clostridia/ Eubacteriales/Lachnospiraceae/****Sellimonas intestinalis*** |  | - | - |  | - | - |  | 0.10 (0.03) | 0.025 |  |  |
| *Actinobacteria/Coriobacteriia/Eggerthellales/Eggerthellaceae/****Eggerthella lenta*** |  | - | - |  | - | - |  | 0.14 (0.05) | 0.026 |  |  |
| *Firmicutes/Erysipelotrichia/Erysipelotrichales/Erysipelotrichaceae/****Erysipelatoclostridium ramosum*** |  | - | - |  | - | - |  | 0.18 (0.06) | 0.021 |  |  |
| *Firmicutes/Clostridia/Lachnospirales/Lachnospiraceae/****Blautia hydrogenotrophica*** |  | -0.07 (0.02) | 0.022 |  | - | - |  | - | - |  |  |
| *Firmicutes/Erysipelotrichia/Erysipelotrichales/Erysipelotrichaceae/****Faecalitalea cylindroides*** |  | -0.10 (0.03) | 0.004 |  | - | - |  | 0.11 (0.03) | 0.001 |  |  |
| *Firmicutes/Clostridia/Eubacteriales/Clostridiaceae/****Clostridium phoceensis*** |  | -0.09 (0.03) | 0.022 |  | - | - |  | - | - |  |  |
| *Firmicutes/Clostridia/Eubacteriales/Lachnospiraceae/****Anaerostipes hadrus*** |  | 0.10 (0.04) | 0.046 |  | - | - |  | - | - |  |  |
|  |  |  |  |  |  |  |  |  |  | | |
| **Cluster 4** |  | 0.04 (0.04) | 0.26 |  | -0.01 (0.04) | 0.86 |  | **-0.11 (0.03)** | **0.001** | | |
| *Bacteroidetes/Bacteroidia/Bacteroidales/Prevotellaceae/****Prevotella brevis*** |  | - | - |  | - | - |  | -0.17 (0.06) | 0.027 |  |  |
| *Bacteroidetes/Bacteroidia/Bacteroidales/Prevotellaceae/****Prevotella multiformis*** |  | - | - |  | - | - |  | -0.11 (0.03) | 0.020 |  |  |
| *Bacteroidetes/Bacteroidia/Bacteroidales/Prevotellaceae/****Prevotella sp. AGR2160*** |  | - | - |  | - | - |  | -0.18 (0.06) | 0.021 |  |  |
| *Bacteroidetes/Bacteroidia/Bacteroidales/Prevotellaceae/****Prevotella nanceiensis*** |  | - | - |  | - | - |  | -0.25 (0.09) | 0.038 |  |  |
|  |  |  |  |  |  |  |  |  |  |  |  |
| **Cluster 5** |  | 0.02 (0.03) | 0.62 |  | 0.01 (0.03) | 0.71 |  | **-0.12 (0.03)** | **<0.001** |  |  |
| *Bacteroidetes/Bacteroidia/Bacteroidales/Rikenellaceae/****Alistipes inops*** |  | - | - |  | - | - |  | -0.20 (0.07) | 0.047 |  |  |
| *Bacteroidetes/Bacteroidia/Bacteroidales/Tannerellaceae/****Parabacteroides distasonis*** |  | - | - |  | - | - |  | -0.15 (0.05) | 0.027 |  |  |
| *Proteobacteria/Betaproteobacteria/Burkholderiales/Sutterellaceae/****Parasutterella excrementihominis*** |  | - | - |  | 0.31 (0.09) | 0.006 |  | - | - |  |  |
| *Bacteroidetes/Bacteroidia/Bacteroidales/Rikenellaceae/****Alistipes shahii*** |  | - | - |  | - | - |  | -0.11 (0.04) | 0.045 |  |  |
| *Bacteroidetes/Bacteroidia/Bacteroidales/Rikenellaceae/****Alistipes obesi*** |  | - | - |  | - | - |  | -0.17 (0.04) | 0.003 |  |  |
|  |  |  |  |  |  |  |  |  |  |  |  |
| **Cluster 6** |  | -0.05 (0.06) | 0.44 |  | -0.10 (0.07) | 0.15 |  | **0.25 (0.06)** | **<0.001** |  |  |
| *Firmicutes/Bacilli/Lactobacillales/Streptococcaceae/****Streptococcus thermophilus*** |  | - | - |  |  |  |  | 0.25 (0.06) | 0.003 |  |  |
| *Firmicutes/Bacilli/Lactobacillales/Lactobacillaceae/****Lactobacillus delbrueckii*** |  | - | - |  |  |  |  | 0.35 (0.09) | 0.003 |  |  |
|  |  |  |  |  |  |  |  |  |  |  |  |
| **Cluster 7** |  | **-0.13 (0.04)** | **0.001** |  | **0.09 (0.04)** | **0.031** |  | **0.12 (0.04)** | **0.003** |  |  |
| *Firmicutes/Bacilli/Lactobacillales/Streptococcaceae/****Streptococcus pneumoniae*** |  | - | - |  | 0.30 (0.07) | 0.001 |  | - | - |  |  |
| *Firmicutes/Bacilli/Lactobacillales/Streptococcaceae/****Streptococcus sanguinis*** |  | -0.21 (0.07) | 0.026 |  | - | - |  | - | - |  |  |
| *Firmicutes/Bacilli/Lactobacillales/Streptococcaceae/****Streptococcus parasanguinis*** |  | -0.16 (0.06) | 0.039 |  | - | - |  | - | - |  |  |
| *Firmicutes/Bacilli/Lactobacillales/Streptococcaceae/****Streptococcus mitis*** |  | -0.16 (0.05) | 0.030 |  | - | - |  | 0.15 (0.05) | 0.033 |  |  |
| *Firmicutes/Bacilli/Lactobacillales/Streptococcaceae/****Streptococcus oralis*** |  | -0.26 (0.07) | 0.003 |  | - | - |  | - | - |  |  |
| *Firmicutes/Bacilli/Lactobacillales/Streptococcaceae/****Streptococcus gordonii*** |  | -0.26 (0.07) | 0.006 |  | - | - |  | - | - |  |  |
| *Firmicutes/Bacilli/Lactobacillales/Streptococcaceae/****Streptococcus infantis*** |  | - | - |  | - | - |  | 0.18 (0.06) | 0.033 |  |  |
| *Firmicutes/Bacilli/Lactobacillales/Streptococcaceae//****Streptococcus salivarius*** |  | -0.19 (0.05) | 0.008 |  | - | - |  | - | - |  |  |
|  |  |  |  |  |  |  |  |  |  |  |  |
| **Cluster 8** |  | **0.18 (0.04)** | **<0.001** |  | -0.03 (0.04) | 0.47 |  | -0.06 (0.04) | 0.17 |  |  |
| *Pseudomonadota/Gammaproteobacteria/Pasteurellales/Pasteurellaceae/ Aggregatibacter aphrophilus* |  | 0.24 (0.09) | 0.043 |  | - | - |  | - | - |  |  |
| *Firmicutes/Clostridia/Eubacteriales/Lachnospiraceae/****[Eubacterium] eligens*** |  | 0.17 (0.04) | 0.002 |  | - | - |  | - | - |  |  |
| *Firmicutes/Clostridia/Eubacteriales/Clostridiaceae/****Clostridium disporicum*** |  | - | - |  | 0.19 (0.06) | 0.026 |  | - | - |  |  |
| *Firmicutes/Clostridia/Eubacteriales/Lachnospiraceae/****Butyrivibrio crossotus*** |  | 0.16 (0.05) | 0.011 |  | - | - |  | - | - |  |  |
|  |  |  |  |  |  |  |  |  |  |  |  |
| **Cluster 9** |  | **0.07 (0.02)** | **0.001** |  | **-0.08 (0.02)** | **0.001** |  | -0.02 (0.02) | 0.30 |  |  |
| *Firmicutes/Clostridia/Eubacteriales/Lachnospiraceae/****Roseburia hominis*** |  | 0.08 (0.02) | 0.012 |  | -0.08 (0.02) | 0.014 |  | - | - |  |  |
| *Firmicutes/Clostridia/Eubacteriales/Lachnospiraceae/****Johnsonella ignava*** |  | - | - |  | -0.07 (0.02) | 0.011 |  | - | - |  |  |
| *Firmicutes/Negativicutes/Selenomonadales/Selenomonadaceae/****Selenomonas ruminantium*** |  | - | - |  | - | - |  | -0.06 (0.02) | 0.026 |  |  |
| *Firmicutes/Clostridia/Eubacteriales/Oscillospiraceae/****Ruminococcus albus*** |  | 0.09 (0.03) | 0.022 |  | - | - |  | - | - |  |  |
| *Firmicutes/Tissierellia/Tissierellales/Peptoniphilaceae/****Peptoniphilus sp. BV3C26*** |  | - | - |  | -0.13 (0.05) | 0.048 |  | - | - |  |  |
|  |  |  |  |  |  |  |  |  |  |  |  |
| **Cluster 10** |  | -0.09 (0.06) | 0.12 |  | -0.06 (0.06) | 0.36 |  | **0.23 (0.06)** | **<0.001** |  |  |
| *Proteobacteria/Gammaproteobacteria/Enterobacterales/Enterobacteriaceae/****Siccibacter turicensis*** |  | - | - |  | - | - |  | 0.21 (0.08) | 0.047 |  |  |
| *Proteobacteria/Gammaproteobacteria/Enterobacterales/Enterobacteriaceae/****Citrobacter amalonaticus*** |  | - | - |  | - | - |  | 0.22 (0.08) | 0.034 |  |  |
| *Proteobacteria/Gammaproteobacteria/Enterobacterales/Enterobacteriaceae/****Klebsiella aerogenes*** |  | - | - |  | - | - |  | 0.25 (0.09) | 0.027 |  |  |
| *Proteobacteria/Gammaproteobacteria/Enterobacterales/Enterobacteriaceae/****Shimwellia blattae*** |  | -0.23 (0.08) | 0.023 |  | - | - |  | 0.22 (0.07) | 0.026 |  |  |
| *Proteobacteria/Gammaproteobacteria/Enterobacterales/Enterobacteriaceae/****Salmonella enterica*** |  | - | - |  | - | - |  | 0.32 (0.08) | 0.002 |  |  |
| *Proteobacteria/Gammaproteobacteria/Enterobacterales/Enterobacteriaceae/****Atlantibacter hermannii*** |  | - | - |  | - | - |  | 0.25 (0.07) | 0.011 |  |  |
| *Proteobacteria/Gammaproteobacteria/Enterobacterales/Enterobacteriaceae/****Klebsiella oxytoca*** |  | - | - |  | - | - |  | 0.31 (0.08) | 0.005 |  |  |
| *Proteobacteria/Gammaproteobacteria/Enterobacterales/Enterobacteriaceae/****Citrobacter freundii*** |  | - | - |  | - | - |  | 0.24 (0.09) | 0.045 |  |  |
| *Proteobacteria/Gammaproteobacteria/Enterobacterales/Enterobacteriaceae/****Enterobacter sp. 638*** |  | - | - |  | - | - |  | 0.26 (0.08) | 0.012 |  |  |
| *Proteobacteria/Gammaproteobacteria/Enterobacterales/Enterobacteriaceae/****Kosakonia sacchari*** |  | - | - |  | - | - |  | 0.23 (0.08) | 0.022 |  |  |
| *¹* Associations between the main food groups and species level taxa were determined with multivariate association with linear models (MaAsLin) tool with centered log-ratio (CLR) –transformed taxa. Benjamini-Hochberg FDR corrected *P* values. The models were adjusted for age, sex, BMI, smoking status, usage of possible microbiome altering medications (metformin and psycholeptics/psychoanaleptics) and total energy intake. | | | | | | | | | |  |  |

| **Supplemental Table 4.** Species-level taxa associations of plant-based foods subgroups. | | | |
| --- | --- | --- | --- |
| **Genus/species** | **Beta** | **SE** | ***P*¹** |
| **Vegetables** |  |  |  |
| *Anaerococcus obesiensis* | 0.21 | 0.07 | 0.030 |
| *Anaerostipes hadrus* | 0.14 | 0.03 | 0.000 |
| *Bacteroides cellulosilyticus* | 0.14 | 0.05 | 0.045 |
| *Bacteroides fluxus* | 0.08 | 0.03 | 0.043 |
| *Bifidobacterium aesculapii* | -0.13 | 0.05 | 0.043 |
| *Bifidobacterium bifidum* | -0.18 | 0.07 | 0.041 |
| *Bifidobacterium breve* | -0.13 | 0.05 | 0.047 |
| *Bifidobacterium dentium* | -0.21 | 0.05 | 0.001 |
| *Bifidobacterium gallinarum* | -0.16 | 0.04 | 0.004 |
| *Bifidobacterium longum* | -0.13 | 0.05 | 0.045 |
| *Bifidobacterium minimum* | -0.18 | 0.06 | 0.042 |
| *Bifidobacterium scardovii* | -0.12 | 0.03 | 0.010 |
| *Bifidobacterium subtile* | -0.23 | 0.06 | 0.001 |
| *Dielma fastidiosa* | 0.17 | 0.06 | 0.040 |
| *Lachnospira multipara* | 0.09 | 0.03 | 0.021 |
| *Parabacteroides distasonis* | 0.15 | 0.05 | 0.023 |
| *Parabacteroides goldsteinii* | 0.13 | 0.04 | 0.012 |
| *Prevotella buccalis* | 0.09 | 0.03 | 0.037 |
| *Prevotella timonensis* | 0.09 | 0.03 | 0.028 |
| *Roseburia faecis* | -0.13 | 0.04 | 0.016 |
| *Shimwellia blattae* | -0.20 | 0.07 | 0.035 |
| *Streptococcus mutans* | -0.26 | 0.07 | 0.003 |
| *Streptococcus oralis* | -0.19 | 0.06 | 0.024 |
| *Streptococcus salivarius* | -0.14 | 0.05 | 0.045 |
| *Turicibacter sanguinis* | -0.19 | 0.07 | 0.039 |
| *[Eubacterium] eligens* | 0.12 | 0.04 | 0.021 |
| **Potatoes** |  |  |  |
| *Enterococcus gilvus* | -0.22 | 0.08 | 0.039 |
| *Luteimonas huabeiensis* | 0.26 | 0.09 | 0.047 |
| **Fruits** |  |  |  |
| *Alistipes putredinis* | -0.14 | 0.05 | 0.033 |
| *Anaerococcus obesiensis* | 0.27 | 0.07 | 0.004 |
| *Bifidobacterium bifidum* | -0.19 | 0.07 | 0.049 |
| *Bittarella massiliensis* | -0.05 | 0.02 | 0.045 |
| *Blautia hydrogenotrophica* | -0.06 | 0.02 | 0.042 |
| *Butyrivibrio crossotus* | 0.14 | 0.05 | 0.023 |
| *Clostridium phoceensis* | -0.09 | 0.03 | 0.012 |
| *Cyanobacteria* | -0.33 | 0.09 | 0.005 |
| *Eubacterium ventriosum* | -0.09 | 0.03 | 0.021 |
| *Gloeobacter violaceus* | 0.29 | 0.09 | 0.023 |
| *Ochrobactrum anthropi* | 0.30 | 0.10 | 0.019 |
| *Roseburia hominis* | 0.06 | 0.02 | 0.031 |
| *Roseburia inulinivorans* | -0.07 | 0.03 | 0.036 |
| *Ruminococcus albus* | 0.10 | 0.03 | 0.007 |
| *Ruminococcus sp. FC2018* | 0.09 | 0.03 | 0.039 |
| *Streptococcus gordonii* | -0.22 | 0.07 | 0.015 |
| *Streptococcus mitis* | -0.14 | 0.05 | 0.036 |
| *Streptococcus mutans* | -0.21 | 0.07 | 0.029 |
| *[Clostridium] spiroforme* | -0.15 | 0.05 | 0.047 |
| *[Eubacterium] eligens* | 0.19 | 0.04 | 0.000 |
| **Cereals** |  |  |  |
| *Actinomyces graevenitzii* | -0.13 | 0.03 | 0.002 |
| *Actinomyces oris* | -0.19 | 0.06 | 0.026 |
| *Adlercreutzia equolifaciens* | -0.17 | 0.05 | 0.010 |
| *Bifidobacterium adolescentis* | 0.40 | 0.07 | 0.000 |
| *Bifidobacterium aesculapii* | 0.19 | 0.06 | 0.011 |
| *Bifidobacterium angulatum* | 0.37 | 0.06 | 0.000 |
| *Bifidobacterium bifidum* | 0.38 | 0.08 | 0.000 |
| *Bifidobacterium breve* | 0.29 | 0.06 | 0.000 |
| *Bifidobacterium callitrichos* | 0.23 | 0.06 | 0.002 |
| *Bifidobacterium choerinum* | 0.26 | 0.06 | 0.001 |
| *Bifidobacterium dentium* | 0.25 | 0.06 | 0.002 |
| *Bifidobacterium gallinarum* | 0.23 | 0.05 | 0.001 |
| *Bifidobacterium longum* | 0.30 | 0.06 | 0.000 |
| *Bifidobacterium minimum* | 0.33 | 0.08 | 0.001 |
| *Bifidobacterium pseudolongum* | 0.19 | 0.06 | 0.010 |
| *Bifidobacterium reuteri* | 0.21 | 0.05 | 0.000 |
| *Bifidobacterium saguini* | 0.27 | 0.06 | 0.000 |
| *Bifidobacterium scardovii* | 0.21 | 0.04 | 0.000 |
| *Bifidobacterium stellenboschense* | 0.11 | 0.04 | 0.018 |
| *Bifidobacterium subtile* | 0.32 | 0.07 | 0.000 |
| *Bifidobacterium thermophilum* | 0.21 | 0.07 | 0.016 |
| *Candidatus Stoquefichus sp. SB1* | -0.17 | 0.05 | 0.006 |
| *Clostridium phoceensis* | -0.09 | 0.03 | 0.033 |
| *Clostridium sp. KLE 1755* | -0.12 | 0.04 | 0.012 |
| *Enterorhabdus mucosicola* | -0.09 | 0.03 | 0.041 |
| *Eubacterium ramulus* | -0.08 | 0.03 | 0.023 |
| *Faecalitalea cylindroides* | -0.08 | 0.03 | 0.035 |
| *Haemophilus influenzae* | 0.31 | 0.10 | 0.020 |
| *Haemophilus sputorum* | 0.28 | 0.10 | 0.031 |
| *Holdemania massiliensis* | -0.12 | 0.04 | 0.011 |
| *Hungatella hathewayi* | -0.08 | 0.02 | 0.017 |
| *Lactobacillus ruminis* | 0.21 | 0.06 | 0.010 |
| *Phocea massiliensis* | -0.09 | 0.02 | 0.007 |
| *Prevotella baroniae* | 0.24 | 0.08 | 0.017 |
| *Prevotella bergensis* | 0.26 | 0.05 | 0.000 |
| *Prevotella dentalis* | 0.15 | 0.05 | 0.039 |
| *Prevotella multisaccharivorax* | 0.23 | 0.06 | 0.003 |
| *Proteiniphilum acetatigenes* | 0.24 | 0.07 | 0.011 |
| *Roseburia faecis* | 0.15 | 0.05 | 0.024 |
| *Ruminococcus faecis* | -0.09 | 0.03 | 0.017 |
| *Ruminococcus sp. AT10* | -0.07 | 0.03 | 0.037 |
| *Streptococcus gordonii* | -0.25 | 0.08 | 0.025 |
| *Streptococcus mitis* | -0.17 | 0.06 | 0.039 |
| *Streptococcus mutans* | -0.27 | 0.08 | 0.018 |
| *Trueperella pyogenes* | -0.23 | 0.06 | 0.002 |
| *Varibaculum cambriense* | -0.23 | 0.08 | 0.043 |
| *[Clostridium] citroniae* | -0.08 | 0.03 | 0.049 |
| *[Clostridium] spiroforme* | -0.18 | 0.06 | 0.037 |
| *[Ruminococcus] torques* | -0.20 | 0.05 | 0.002 |
| ¹ Associations between each plant-based food subgroup and species level taxa were determined with multivariate association with linear models (MaAsLin) tool with centered log-ratio (CLR) –transformed taxa. Benjamini-Hochberg FDR corrected *P* values. The models were adjusted for age, sex, BMI, smoking status, usage of possible microbiome altering medications (metformin and psycholeptics/psychoanaleptics) and total energy intake. | | | |

| **Supplemental Table 5.** Species-level taxa associations of red and processed meat subgroups. | | | |
| --- | --- | --- | --- |
| **Genus/species** | **Beta** | **SE** | ***P*¹** |
| **Red meat** |  |  |  |
| *Cellulomonas carbonis* | -0.23 | 0.08 | 0.038 |
| *Parasutterella excrementihominis* | 0.24 | 0.08 | 0.023 |
| *Porphyromonas somerae* | -0.19 | 0.05 | 0.006 |
| *Prevotella bergensis* | -0.15 | 0.05 | 0.013 |
| *Prevotella oris* | -0.22 | 0.07 | 0.017 |
| **Processed meat** |  |  |  |
| *Johnsonella ignava* | -0.07 | 0.02 | 0.010 |
| *Streptococcus pneumoniae* | 0.22 | 0.06 | 0.010 |
| *Coprococcus sp. HPP0048* | 0.09 | 0.03 | 0.011 |
| *Roseburia hominis* | -0.07 | 0.02 | 0.017 |
| *Anaerostipes hadrus* | -0.11 | 0.04 | 0.021 |
| *Dorea sp. 5.2* | -0.07 | 0.02 | 0.021 |
| *[Clostridium] viride* | -0.07 | 0.02 | 0.034 |
| ¹ Associations between the red and processed meat subgroups and species level taxa were determined with multivariate association with linear models (MaAsLin) tool with centered log-ratio (CLR) –transformed taxa. Benjamini-Hochberg FDR corrected *P* values. The models were adjusted for age, sex, BMI, smoking status, usage of possible microbiome altering medications (metformin and psycholeptics/psychoanaleptics) and total energy intake. | | | |

| **Supplemental Table 6.** Species-level taxa associations of dairy subgroups. | | | |
| --- | --- | --- | --- |
| **Genus/species** | **Beta** | **SE** | ***P*¹** |
| **Fermented milk** |  |  |  |
| *Bifidobacterium animalis* | 0.28 | 0.06 | 0.031 |
| *Bifidobacterium longum* | 0.15 | 0.05 | 0.031 |
| *Kocuria sp. ZOR0020* | 0.71 | 0.07 | 0.031 |
| *Lactobacillus acidophilus* | 0.35 | 0.07 | 0.032 |
| *Lactobacillus delbrueckii* | 0.51 | 0.08 | 0.032 |
| *Lactobacillus rhamnosus* | 0.41 | 0.09 | 0.032 |
| *Lactococcus lactis* | 0.47 | 0.08 | 0.032 |
| *Leuconostoc mesenteroides* | 1.15 | 0.08 | 0.032 |
| *Leuconostoc pseudomesenteroides* | 0.49 | 0.06 | 0.032 |
| *Megasphaera cerevisiae* | -0.20 | 0.07 | 0.032 |
| *Methylobacterium sp. ZNC0032* | 0.56 | 0.08 | 0.033 |
| *Prevotella nanceiensis* | -0.25 | 0.08 | 0.033 |
| *Prevotella nigrescens* | -0.23 | 0.08 | 0.033 |
| *Prevotella ruminicola* | -0.19 | 0.06 | 0.034 |
| *Streptococcus thermophilus* | 0.40 | 0.06 | 0.034 |
| **Cheese** |  |  |  |
| *Anaerostipes hadrus* | 0.12 | 0.03 | 0.005 |
| *Blautia obeum* | 0.07 | 0.02 | 0.045 |
| *Butyrivibrio proteoclasticus* | 0.07 | 0.02 | 0.028 |
| *Catonella morbi* | 0.07 | 0.02 | 0.033 |
| *Dorea longicatena* | 0.09 | 0.03 | 0.017 |
| *Eubacterium sp. 14.2* | 0.07 | 0.02 | 0.016 |
| *Eubacterium xylanophilum* | 0.11 | 0.03 | 0.006 |
| *Kocuria sp. ZOR0020* | 0.26 | 0.08 | 0.013 |
| *Lachnobacterium bovis* | 0.08 | 0.03 | 0.022 |
| *Lachnospira multipara* | 0.09 | 0.03 | 0.021 |
| *Lactococcus lactis* | 0.67 | 0.08 | 0.000 |
| *Methylobacterium sp. ZNC0032* | 0.27 | 0.08 | 0.012 |
| *Ruminococcus bicirculans* | 0.15 | 0.06 | 0.044 |
| *Ruminococcus gauvreauii* | 0.05 | 0.02 | 0.044 |
| *Shuttleworthia satelles* | 0.05 | 0.02 | 0.035 |
| *[Clostridium] saccharogumia* | 0.11 | 0.04 | 0.032 |
| **Other dairy products** |  |  |  |
| *Actinomyces oris* | 0.18 | 0.05 | 0.012 |
| *Alistipes ihumii* | -0.17 | 0.06 | 0.049 |
| *Alistipes obesi* | -0.17 | 0.04 | 0.003 |
| *Alistipes putredinis* | -0.15 | 0.05 | 0.028 |
| *Alistipes timonensis* | -0.13 | 0.04 | 0.032 |
| *Atlantibacter hermannii* | 0.25 | 0.07 | 0.013 |
| *Enterobacter cloacae* | 0.22 | 0.08 | 0.045 |
| *Enterobacter sp. 638* | 0.26 | 0.08 | 0.011 |
| *Erysipelatoclostridium ramosum* | 0.19 | 0.06 | 0.013 |
| *Faecalitalea cylindroides* | 0.08 | 0.03 | 0.024 |
| *Klebsiella oxytoca* | 0.29 | 0.08 | 0.008 |
| *Klebsiella sp. RIT PI d* | 0.21 | 0.08 | 0.044 |
| *Kosakonia sacchari* | 0.25 | 0.08 | 0.012 |
| *Lachnospira multipara* | -0.10 | 0.03 | 0.012 |
| *Megamonas funiformis* | -0.20 | 0.07 | 0.039 |
| *Methylobacterium sp. ZNC0032* | -0.29 | 0.08 | 0.008 |
| *Oscillibacter sp. ER4* | -0.09 | 0.03 | 0.050 |
| *Parabacteroides distasonis* | -0.15 | 0.05 | 0.027 |
| *Prevotella multiformis* | -0.10 | 0.03 | 0.041 |
| *Prevotella sp. AGR2160* | -0.16 | 0.06 | 0.042 |
| *Pseudomonas knackmussii* | 0.32 | 0.10 | 0.012 |
| *Salmonella enterica* | 0.29 | 0.08 | 0.005 |
| *Selenomonas ruminantium* | -0.07 | 0.02 | 0.006 |
| *Sellimonas intestinalis* | 0.09 | 0.03 | 0.033 |
| *Shimwellia blattae* | 0.23 | 0.07 | 0.018 |
| *Siccibacter turicensis* | 0.21 | 0.08 | 0.046 |
| *Streptococcus anginosus* | 0.28 | 0.08 | 0.007 |
| *Streptococcus parasanguinis* | 0.16 | 0.06 | 0.032 |
| *Streptococcus salivarius* | 0.15 | 0.05 | 0.045 |
| *[Clostridium] viride* | -0.07 | 0.02 | 0.049 |
| *[Eubacterium] eligens* | -0.13 | 0.04 | 0.028 |
| ¹ Associations between each dairy subgroup and species level taxa were determined with multivariate association with linear models (MaAsLin) tool with centered log-ratio (CLR) –transformed taxa. Benjamini-Hochberg FDR corrected *P* values. The models were adjusted for age, sex, BMI, smoking status, usage of possible microbiome altering medications (metformin and psycholeptics/psychoanaleptics) and total energy intake. | | | |

| **Supplemental Table 7.** Significant results (BH corrected *P*-value <0.05) of pathway analysis. | | | | | |
| --- | --- | --- | --- | --- | --- |
| **KO-group** | **Beta** | **SE** | ***P*-value BH cor** | **Name** | **Pathway** |
| **Dairy** |  |  |  |  |  |
| K00013 | -1.9E-05 | 5.20E-06 | 0.03 | histidinol dehydrogenase | Histidine metabolism, Metabolic pathways, Biosynthesis of secondary metabolites, Biosynthesis of amino acids |
| K00145 | -1.9E-05 | 5.40E-06 | 0.03 | N-acetyl-gamma-glutamyl-phosphate reductase | Arginine biosynthesis, Metabolic pathways, Biosynthesis of secondary metabolites, 2-Oxocarboxylic acid metabolism, Biosynthesis of amino acids |
| K00215 | -1.8E-05 | 5.30E-06 | 0.04 | 4-hydroxy tetrahydrodipicolinate reductase | Monobactam biosynthesis, Lysine biosynthesis, Metabolic pathways, Biosynthesis of secondary metabolites, Microbial metabolism in diverse environments, Biosynthesis of amino acids |
| K00262 | -1.7E-05 | 4.60E-06 | 0.03 | glutamate dehydrogenase (NADP+) | Arginine biosynthesis, Alanine, aspartate and glutamate metabolism, Nitrogen metabolism, Metabolic pathways, Microbial metabolism in diverse environments |
| K00278 | -2.5E-05 | 6.70E-06 | 0.02 | L-aspartate oxidase | Alanine, aspartate and glutamate metabolism, Nicotinate and nicotinamide metabolism, Metabolic pathways, Biosynthesis of cofactors |
| K00290 | -4.4E-05 | 0.000011 | 0.02 | saccharopine dehydrogenase (NAD+, L-lysine forming) | Lysine biosynthesis, Lysine degradation,  Metabolic pathways, Biosynthesis of secondary metabolites, Biosynthesis of amino acids |
| K00364 | 0.00024 | 0.00006 | 0.02 | GMP reductase | Purine metabolism, Metabolic pathways,  Nucleotide metabolism |
| K00383 | 0.00021 | 0.00005 | 0.02 | glutathione reductase (NADPH) | Glutathione metabolism, Metabolic pathways, Thyroid hormone synthesis,  Diabetic cardiomyopathy |
| K00563 | 0.00018 | 0.00005 | 0.04 | 23S rRNA (guanine745-N1)-methyltransferase |  |
| K00564 | 0.0002 | 0.00005 | 0.02 | 16S rRNA (guanine1207-N2)-methyltransferase |  |
| K00687 | 0.00022 | 0.00007 | 0.04 | penicillin-binding protein 2B | Peptidoglycan biosynthesis, Metabolic pathways, beta-Lactam resistance |
| K00767 | -2.3E-05 | 5.90E-06 | 0.02 | nicotinate-nucleotide pyrophosphorylase (carboxylating) | Nicotinate and nicotinamide metabolism,  Metabolic pathways, Biosynthesis of cofactors |
| K00793 | -2.5E-05 | 6.50E-06 | 0.02 | riboflavin synthase | Riboflavin metabolism, Metabolic pathways,  Biosynthesis of secondary metabolites, Biosynthesis of cofactors |
| K00794 | -3.2E-05 | 8.10E-06 | 0.02 | 6,7-dimethyl-8-ribityllumazine synthase | Riboflavin metabolism, Metabolic pathways,  Biosynthesis of secondary metabolites,  Biosynthesis of cofactors |
| K00867 | 0.00021 | 0.00006 | 0.03 | type I pantothenate kinase | Pantothenate and CoA biosynthesis, Metabolic pathways, Biosynthesis of cofactors |
| K00946 | -3.8E-05 | 0.00001 | 0.03 | thiamine-monophosphate kinase | Thiamine metabolism, Metabolic pathways,  Biosynthesis of cofactors |
| K00989 | 0.00017 | 0.00005 | 0.04 | ribonuclease PH |  |
| K01002 | 0.00018 | 0.00005 | 0.03 | phosphoglycerol transferase |  |
| K01006 | -4.1E-05 | 0.000011 | 0.02 | pyruvate, orthophosphate dikinase | Glycolysis / Gluconeogenesis, Pyruvate metabolism, Carbon fixation in photosynthetic organisms, Carbon fixation pathways in prokaryotes, Metabolic pathways, Biosynthesis of secondary metabolites, Microbial metabolism in diverse environments, Carbon metabolism |
| K01151 | -2.3E-05 | 5.70E-06 | 0.02 | deoxyribonuclease IV | Base excision repair |
| K01226 | 0.00021 | 0.00006 | 0.02 | trehalose-6-phosphate hydrolase | Starch and sucrose metabolism, Metabolic pathways |
| K01261 | 0.00016 | 0.00005 | 0.04 | glutamyl aminopeptidase |  |
| K01271 | 0.00023 | 0.00005 | 0.02 | Xaa-Pro dipeptidase |  |
| K01361 | 0.00019 | 0.00006 | 0.04 | lactocepin |  |
| K01467 | 0.00022 | 0.00006 | 0.03 | beta-lactamase class C | beta-Lactam resistance, Two-component system |
| K01479 | 0.00018 | 0.00006 | 0.04 | formiminoglutamase | Histidine metabolism, Metabolic pathways |
| K01537 | -3.3E-05 | 9.90E-06 | 0.04 | P-type Ca2+ transporter type 2C |  |
| K01731 | 0.00033 | 0.0001 | 0.05 | pectate disaccharide-lyase | Pentose and glucuronate interconversions,  Metabolic pathways |
| K01772 | 0.0002 | 0.00005 | 0.02 | protoporphyrin/coproporphyrin ferrochelatase | Porphyrin metabolism, Metabolic pathways, Biosynthesis of secondary metabolites, Biosynthesis of cofactors |
| K01787 | -4.9E-05 | 0.000014 | 0.04 | N-acylglucosamine 2-epimerase | Amino sugar and nucleotide sugar metabolism, Metabolic pathways |
| K01788 | 0.00016 | 0.00004 | 0.03 | N-acylglucosamine-6-phosphate 2-epimerase | Amino sugar and nucleotide sugar metabolism, Metabolic pathways |
| K01814 | -2.4E-05 | 6.30E-06 | 0.02 | phosphoribosylformimino-5-aminoimidazole carboxamide ribotide isomerase | Histidine metabolism, Metabolic pathways, Biosynthesis of secondary metabolites, Biosynthesis of amino acids |
| K01878 | 0.00017 | 0.00005 | 0.04 | glycyl-tRNA synthetase alpha chain | Aminoacyl-tRNA biosynthesis |
| K01918 | -2.7E-05 | 7.00E-06 | 0.02 | pantoate--beta-alanine ligase | beta-Alanine metabolism, Pantothenate and CoA biosynthesis, Metabolic pathways, Biosynthesis of secondary metabolites, Biosynthesis of cofactors |
| K01950 | -0.00006 | 0.000011 | <0.001 | NAD+ synthase (glutamine-hydrolysing) | Nicotinate and nicotinamide metabolism, Metabolic pathways, Biosynthesis of cofactors |
| K02082 | 0.00021 | 0.00006 | 0.04 | D-galactosamine 6-phosphate deaminase/isomerase | Galactose metabolism, Metabolic pathways |
| K02346 | -0.00002 | 5.80E-06 | 0.04 | DNA polymerase IV |  |
| K02428 | -2.2E-05 | 6.60E-06 | 0.04 |  |  |
| K02744 | 0.00014 | 0.00004 | 0.05 | N-acetylgalactosamine PTS system EIIA component | Galactose metabolism, Metabolic pathways, Phosphotransferase system (PTS) |
| K02768 | 0.00017 | 0.00005 | 0.04 | fructose PTS system EIIA component | Fructose and mannose metabolism, Metabolic pathways, Microbial metabolism in diverse environments, Phosphotransferase system (PTS) |
| K02769 | 0.00018 | 0.00005 | 0.03 | fructose PTS system EIIB component | Fructose and mannose metabolism, Metabolic pathways, Microbial metabolism in diverse environments, Phosphotransferase system (PTS) |
| K02794 | 0.00015 | 0.00005 | 0.04 | mannose PTS system EIIAB component | Fructose and mannose metabolism, Amino sugar and nucleotide sugar metabolism, Metabolic pathways, Phosphotransferase system (PTS) |
| K03081 | 0.00024 | 0.00007 | 0.04 |  |  |
| K03366 | 0.00018 | 0.00005 | 0.04 | meso-butanediol dehydrogenase / (S,S)-butanediol dehydrogenase / diacetyl reductase | Butanoate metabolism,  Biosynthesis of secondary metabolites |
| K03367 | 0.0002 | 0.00006 | 0.03 | D-alanine--poly(phosphoribitol) ligase subunit 1 | D-Amino acid metabolism, Teichoic acid biosynthesis, Metabolic pathways,  Cationic antimicrobial peptide (CAMP) resistance, Two-component system,  Staphylococcus aureus infection |
| K03517 | -2.3E-05 | 0.000006 | 0.02 | quinolinate synthase | Nicotinate and nicotinamide metabolism, Metabolic pathways, Biosynthesis of cofactors |
| K03527 | -2.1E-05 | 5.80E-06 | 0.03 | 4-hydroxy-3-methylbut-2-en-1-yl diphosphate reductase | Terpenoid backbone biosynthesis, Metabolic pathways, Biosynthesis of secondary metabolites |
| K03551 | -1.7E-05 | 5.40E-06 | 0.05 | holliday junction DNA helicase RuvB | Homologous recombination |
| K03830 | 0.00016 | 0.00005 | 0.04 | putative acetyltransferase |  |
| K05362 | 0.00021 | 0.00006 | 0.04 | UDP-N-acetylmuramoyl-L-alanyl-D-glutamate-L-lysine ligase | Peptidoglycan biosynthesis, Metabolic pathways |
| K05363 | 0.00014 | 0.00004 | 0.05 | serine/alanine adding enzyme | Peptidoglycan biosynthesis, Metabolic pathways |
| K06001 | -4.8E-05 | 0.000012 | 0.02 | tryptophan synthase beta chain | Glycine, serine and threonine metabolism, Phenylalanine, tyrosine and tryptophan biosynthesis, Metabolic pathways, Biosynthesis of secondary metabolites, Biosynthesis of amino acids |
| K06193 | 0.00021 | 0.00005 | 0.02 | protein PhnA |  |
| K06726 | 0.00018 | 0.00005 | 0.04 | D-ribose pyranase | ABC transporters |
| K06969 | -3.9E-05 | 0.000012 | 0.04 | 23S rRNA (cytosine1962-C5)-methyltransferase |  |
| K07107 | -0.00003 | 8.80E-06 | 0.04 | acyl-CoA thioester hydrolase |  |
| K07636 | -2.5E-05 | 7.50E-06 | 0.04 | two-component system, OmpR family, phosphate regulon sensor histidine kinase PhoR | two-component system |
| K08298 | 0.00019 | 0.00006 | 0.05 | L-carnitine CoA-transferase |  |
| K08303 | -0.00002 | 5.80E-06 | 0.04 | U32 family peptidase | Epithelial cell signaling in Helicobacter pylori infection |
| K08728 | 0.00037 | 0.00009 | 0.02 | nucleoside deoxyribosyltransferase |  |
| K08961 | -0.00019 | 0.000056 | 0.04 | chondroitin-sulfate-ABC endolyase/exolyase |  |
| K09994 | 0.00018 | 0.00005 | 0.03 | (aminoalkyl)phosphonate N-acetyltransferase | Phosphonate and phosphinate metabolism |
| K12453 | -0.00019 | 0.000056 | 0.04 | CDP-paratose synthetase | Amino sugar and nucleotide sugar metabolism, O-Antigen nucleotide sugar biosynthesis, Metabolic pathways, Biosynthesis of nucleotide sugars |
| K12555 | 0.00019 | 0.00006 | 0.04 | penicillin-binding protein 2A | Peptidoglycan biosynthesis, Metabolic pathways, beta-Lactam resistance |
| K12556 | 0.00023 | 0.00007 | 0.04 | penicillin-binding protein 2X | Peptidoglycan biosynthesis, Metabolic pathways, beta-Lactam resistance |
| K12700 | 0.00023 | 0.00006 | 0.02 | non-specific riboncleoside hydrolase | Pyrimidine metabolism, Metabolic pathways, Nucleotide metabolism |
| K13000 | 0.0003 | 0.00008 | 0.03 | mannosyltransferase |  |
| K13498 | 0.00026 | 0.00007 | 0.02 | indole-3-glycerol phosphate synthase / phosphoribosylanthranilate isomerase | Phenylalanine, tyrosine and tryptophan biosynthesis, Metabolic pathways, Biosynthesis of secondary metabolites, Biosynthesis of amino acids |
| K13747 | -4.4E-05 | 0.000011 | 0.02 | carboxynorspermidine decarboxylase | carboxyspermidine carboxy-lyase (spermidine-forming), carboxynorspermidine carboxy-lyase (norspermidine-forming) |
| K13786 | -0.00035 | 0.000104 | 0.04 | cob(II)yrinic acid a,c-diamide reductase | Porphyrin metabolism, Metabolic pathways |
| K13953 | 0.0002 | 0.00006 | 0.03 | alcohol dehydrogenase, propanol-preferring | Glycolysis / Gluconeogenesis, Fatty acid degradation, Tyrosine metabolism, Pyruvate metabolism, Chloroalkane and chloroalkene degradation, Naphthalene degradation, Retinol metabolism, Metabolism of xenobiotics by cytochrome P450, Drug metabolism - cytochrome P450, Metabolic pathways, Biosynthesis of secondary metabolites, Microbial metabolism in diverse environments, Degradation of aromatic compounds |
| K14441 | -2.5E-05 | 7.50E-06 | 0.04 | ribosomal protein S12 methylthiotransferase |  |
| K14652 | -3.7E-05 | 0.000011 | 0.03 | 3,4-dihydroxy 2-butanone 4-phosphate synthase / GTP cyclohydrolase II | Riboflavin metabolism, Folate biosynthesis, Metabolic pathways, Biosynthesis of secondary metabolites, Biosynthesis of cofactors |
| K14982 | 0.00017 | 0.00005 | 0.03 | two-component system, OmpR family, sensor histidine kinase CiaH | Two-component system, Quorum sensing |
| K15633 | -2.4E-05 | 0.000007 | 0.03 | 2,3-bisphosphoglycerate-independent phosphoglycerate mutase | Glycolysis / Gluconeogenesis, Glycine, serine and threonine metabolism, Methane metabolism, Metabolic pathways, Biosynthesis of secondary metabolites, Microbial metabolism in diverse environments, Carbon metabolism, Biosynthesis of amino acids |
| **Fermented milk** | | |  |  |  |
| K00034 | 0.00032 | 9.82E-05 | 0.040 | glucose 1-dehydrogenase | Pentose phosphate pathway, Metabolic pathways, Microbial metabolism in diverse environments, Carbon metabolism |
| K00054 | 0.000677 | 0.000123 | <0.001 | hydroxymethylglutaryl-CoA reductase | Terpenoid backbone biosynthesis, Biosynthesis of secondary metabolites |
| K00055 | 0.000531 | 0.000116 | <0.001 | aryl-alcohol dehydrogenase | Tyrosine metabolism, Phenylalanine metabolism, Xylene degradation, Toluene degradation, Metabolic pathways, Microbial metabolism in diverse environments, Degradation of aromatic compounds |
| K00068 | 0.000581 | 0.000158 | <0.001 | sorbitol-6-phosphate 2-dehydrogenase | Fructose and mannose metabolism, Metabolic pathways |
| K00105 | 0.000526 | 0.000118 | <0.001 | alpha-glycerophosphate oxidase | Glycerophospholipid metabolism, Biosynthesis of secondary metabolites |
| K00131 | 0.000681 | 0.000117 | <0.001 | glyceraldehyde-3-phosphate dehydrogenase (NADP+) | Glycolysis / Gluconeogenesis,  Pentose phosphate pathway, Metabolic pathways, Microbial metabolism in diverse environments, Carbon metabolism |
| K00158 | 0.000436 | 0.000112 | 0.005 | pyruvate oxidase | Pyruvate metabolism, Metabolic pathways |
| K00298 | 0.000755 | 0.000226 | 0.031 | N5-(carboxyethyl)ornithine synthase |  |
| K00383 | 0.000558 | 0.000115 | <0.001 | glutathione reductase (NADPH) | Glutathione metabolism, Metabolic pathways, Thyroid hormone synthesis, Diabetic cardiomyopathy |
| K00433 | 0.000656 | 0.000107 | <0.001 | non-heme chloroperoxidase |  |
| K00563 | 0.000502 | 0.00011 | <0.001 | 23S rRNA (guanine745-N1)-methyltransferase |  |
| K00564 | 0.000431 | 0.000102 | 0.001 | 16S rRNA (guanine1207-N2)-methyltransferase |  |
| K00687 | 0.00078 | 0.000137 | <0.001 | penicillin-binding protein 2B | Peptidoglycan biosynthesis, Metabolic pathways, beta-Lactam resistance |
| K00712 | 0.001025 | 0.000218 | <0.001 | poly(glycerol-phosphate) alpha-glucosyltransferase |  |
| K00756 | 0.00036 | 0.000113 | 0.049 | pyrimidine-nucleoside phosphorylase | Pyrimidine metabolism, Metabolic pathways, Nucleotide metabolism |
| K00841 | 0.00054 | 0.000112 | <0.001 | aminotransferase | Lysine biosynthesis, Metabolic pathways, Biosynthesis of secondary metabolites, Biosynthesis of amino acids |
| K00867 | 0.000575 | 0.000125 | <0.001 | type I pantothenate kinase | Pantothenate and CoA biosynthesis, Metabolic pathways, Biosynthesis of cofactors |
| K00869 | 0.00073 | 0.000136 | <0.001 | mevalonate kinase | Terpenoid backbone biosynthesis, Metabolic pathways, Biosynthesis of secondary metabolites, Peroxisome |
| K00938 | 0.000815 | 0.000126 | <0.001 | phosphomevalonate kinase | Terpenoid backbone biosynthesis, Metabolic pathways, Biosynthesis of secondary metabolites |
| K00984 | 0.000499 | 0.000111 | <0.001 | streptomycin 3"-adenylyltransferase |  |
| K01071 | 0.000819 | 0.000131 | <0.001 | medium-chain acyl-[acyl-carrier-protein] hydrolase | Fatty acid biosynthesis, Metabolic pathways |
| K01101 | 0.000791 | 0.000102 | <0.001 | 4-nitrophenyl phosphatase | Aminobenzoate degradation, Microbial metabolism in diverse environments |
| K01226 | 0.000528 | 0.000116 | 0.000 | trehalose-6-phosphate hydrolase | Starch and sucrose metabolism, Metabolic pathways |
| K01261 | 0.000438 | 9.48E-05 | 0.000 | glutamyl aminopeptidase |  |
| K01271 | 0.000537 | 0.000113 | 0.000 | Xaa-Pro dipeptidase |  |
| K01274 | 0.00048 | 0.000122 | <0.001 | beta-Ala-Xaa dipeptidase |  |
| K01281 | 0.000817 | 0.000131 | <0.001 | X-Pro dipeptidyl-peptidase |  |
| K01361 | 0.000637 | 0.000122 | <0.001 | lactocepin |  |
| K01467 | 0.000527 | 0.000133 | 0.004 | beta-lactamase class C | beta-Lactam resistance, Two-component system |
| K01488 | 0.00034 | 9.53E-05 | 0.016 | adenosine deaminase | Purine metabolism, Metabolic pathways, Nucleotide metabolism, Primary immunodeficiency |
| K01574 | 0.000486 | 0.000106 | <0.001 | acetoacetate decarboxylase | Butanoate metabolism, Metabolic pathways |
| K01597 | 0.000744 | 0.000125 | <0.001 | diphosphomevalonate decarboxylase | Terpenoid backbone biosynthesis, Metabolic pathways, Biosynthesis of secondary metabolites |
| K01641 | 0.000542 | 0.000132 | 0.002 | hydroxymethylglutaryl-CoA synthase | Valine, leucine and isoleucine, Butanoate metabolism, Terpenoid backbone biosynthesis, Metabolic pathways, Biosynthesis of secondary metabolites, PPAR signaling pathway |
| K01674 | 0.001139 | 0.000125 | <0.001 | carbonic anhydrase | Nitrogen metabolism, Metabolic pathways |
| K01772 | 0.000388 | 9.56E-05 | 0.003 | protoporphyrin/coproporphyrin ferrochelatase | Porphyrin metabolism, Metabolic pathways,  Biosynthesis of secondary metabolites,  Biosynthesis of cofactors |
| K01916 | 0.000388 | 0.00012 | 0.044 | NAD+ synthase | Nicotinate and nicotinamide metabolism, Metabolic pathways, Biosynthesis of cofactors |
| K01962 | 0.000331 | 9.78E-05 | 0.028 | acetyl-CoA carboxylase carboxyl transferase subunit alpha | Fatty acid biosynthesis, Pyruvate metabolism, Propanoate metabolism, Carbon fixation pathways in prokaryotes, Metabolic pathways, Biosynthesis of secondary metabolites, Microbial metabolism in diverse environments, Carbon metabolism, Fatty acid metabolism |
| K02204 | 0.000338 | 9.52E-05 | 0.016 | homoserine kinase type II | Glycine, serine and threonine metabolism, Metabolic pathways, Biosynthesis of secondary metabolites, Microbial metabolism in diverse environments, Biosynthesis of amino acids |
| K02756 | 0.001188 | 0.000235 | <0.001 |  |  |
| K02768 | 0.000344 | 0.000102 | 0.028 | fructose PTS system EIIA component | Fructose and mannose metabolism, Metabolic pathways, Microbial metabolism in diverse environments, Phosphotransferase system (PTS) |
| K02769 | 0.000343 | 0.000105 | 0.039 | fructose PTS system EIIB component | Fructose and mannose metabolism, Metabolic pathways, Microbial metabolism in diverse environments, Phosphotransferase system (PTS) |
| K02773 | 0.000429 | 0.000125 | 0.025 | galactitol PTS system EIIA component | Galactose metabolism, Metabolic pathways,  Phosphotransferase system (PTS) |
| K02777 | 0.000417 | 0.000128 | 0.040 | sugar PTS system EIIA component | Glycolysis / Gluconeogenesis, Starch and sucrose metabolism, Amino sugar and nucleotide sugar metabolism, Metabolic pathways, Biofilm formation - Escherichia coli, Phosphotransferase system (PTS), Biofilm formation - Vibrio cholerae |
| K02781 | 0.000494 | 0.000133 | 0.010 | glucitol/sorbitol PTS system EIIA component | Fructose and mannose metabolism, Metabolic pathways, Phosphotransferase system (PTS) |
| K02794 | 0.000322 | 9.42E-05 | 0.026 | mannose PTS system EIIAB component | Fructose and mannose metabolism, Amino sugar and nucleotide sugar metabolism, Metabolic pathways, Phosphotransferase system (PTS) |
| K03081 | 0.000645 | 0.000144 | 0.000 |  |  |
| K03339 | 0.000729 | 0.000144 | <0.001 | 6-phospho-5-dehydro-2-deoxy-D-gluconate aldolase | Inositol phosphate metabolism, Metabolic pathways, Microbial metabolism in diverse environments |
| K03342 | 0.000626 | 0.000119 | <0.001 | para-aminobenzoate synthetase / 4-amino-4-deoxychorismate lyase | Folate biosynthesis, Biosynthesis of cofactors |
| K03366 | 0.000713 | 0.000107 | <0.001 | meso-butanediol dehydrogenase / (S,S)-butanediol dehydrogenase / diacetyl reductase | Butanoate metabolism, Biosynthesis of secondary metabolites |
| K03367 | 0.000587 | 0.000114 | <0.001 | D-alanine--poly(phosphoribitol) ligase subunit 1 | D-Amino acid metabolism, Teichoic acid biosynthesis, Metabolic pathways, Cationic antimicrobial peptide (CAMP) resistance, Two-component system, Staphylococcus aureus infection |
| K03471 | 0.000581 | 0.000137 | 0.001 | ribonuclease HIII | DNA replication |
| K03828 | 0.000611 | 0.000139 | 0.001 | putative acetyltransferase |  |
| K03930 | 0.000444 | 0.000119 | 0.008 | putative tributyrin esterase |  |
| K04782 | 0.000383 | 0.000104 | 0.010 | isochorismate pyruvate lyase | Biosynthesis of siderophore group nonribosomal peptides, Biosynthesis of secondary metabolites |
| K05362 | 0.000745 | 0.000133 | <0.001 | UDP-N-acetylmuramoyl-L-alanyl-D-glutamate-L-lysine ligase | Peptidoglycan biosynthesis, Metabolic pathways |
| K05363 | 0.000369 | 9.14E-05 | 0.003 | serine/alanine adding enzyme | Peptidoglycan biosynthesis, Metabolic pathways |
| K05823 | 0.000752 | 0.00013 | <0.001 | N-acetyldiaminopimelate deacetylase | Lysine biosynthesis, Metabolic pathways,  Biosynthesis of secondary metabolites,  Biosynthesis of amino acids |
| K05910 | 0.001205 | 0.000186 | <0.001 | NADH peroxidase |  |
| K05964 | 0.000516 | 0.000144 | 0.016 | holo-ACP synthase | Two-component system |
| K06193 | 0.000389 | 0.000115 | 0.028 | protein PhnA |  |
| K06606 | 0.000585 | 0.000118 | <0.001 | 2-keto-myo-inositol isomerase | Inositol phosphate metabolism, Metabolic pathways, Microbial metabolism in diverse environments |
| K07305 | 0.000375 | 0.000111 | 0.028 | peptide-methionine (R)-S-oxide reductase |  |
| K07652 | 0.000584 | 0.000111 | <0.001 | two-component system, OmpR family, sensor histidine kinase VicK | Two-component system |
| K07778 | 0.00067 | 0.00011 | <0.001 | two-component system, NarL family, sensor histidine kinase DesK | Two-component system |
| K08317 | 0.000536 | 0.000158 | 0.028 | hydroxycarboxylate dehydrogenase A |  |
| K08728 | 0.002048 | 0.000178 | 7.64E-26 | nucleoside deoxyribosyltransferase |  |
| K08969 | 0.00054 | 0.000139 | 0.005 | L-glutamine---4-(methylsulfanyl)-2-oxobutanoate aminotransferase | Cysteine and methionine metabolism, Metabolic pathways |
| K10353 | 0.001173 | 0.00024 | <0.001 | deoxyadenosine kinase | Purine metabolism, Metabolic pathways, Nucleotide metabolism |
| K10831 | 0.000475 | 0.000144 | 0.036 | taurine transport system ATP-binding protein | Sulfur metabolism, ABC transporters |
| K11617 | 0.000667 | 0.000124 | <0.001 | two-component system, NarL family, sensor histidine kinase LiaS | Two-component system |
| K12111 | 0.000425 | 0.000131 | 0.043 | evolved beta-galactosidase subunit alpha | Galactose metabolism, Other glycan degradation, Metabolic pathways |
| K12554 | 0.000879 | 0.000118 | <0.001 | alanine adding enzyme | Peptidoglycan biosynthesis, Metabolic pathways |
| K12555 | 0.000889 | 0.000116 | <0.001 | penicillin-binding protein 2A | Peptidoglycan biosynthesis, Metabolic pathways, beta-Lactam resistance |
| K12556 | 0.000773 | 0.000137 | <0.001 | penicillin-binding protein 2X | Peptidoglycan biosynthesis, Metabolic pathways, beta-Lactam resistance |
| K12700 | 0.000457 | 0.000127 | 0.014 | non-specific riboncleoside hydrolase | Pyrimidine metabolism, Metabolic pathways, Nucleotide metabolism |
| K12990 | 0.000488 | 0.000116 | 0.001 | rhamnosyltransferase | Quorum sensing, Biofilm formation - Pseudomonas aeruginosa |
| K12996 | 0.000612 | 0.000128 | 0.000 | rhamnosyltransferase |  |
| K12997 | 0.000702 | 0.000129 | <0.001 | rhamnosyltransferase |  |
| K12999 | 0.001315 | 0.000196 | <0.001 | glucosyltransferase |  |
| K13677 | 0.000536 | 9.93E-05 | <0.001 | 1,2-diacylglycerol-3-alpha-glucose alpha-1,2-glucosyltransferase | Glycerolipid metabolism, Metabolic pathways |
| K13678 | 0.000751 | 0.000135 | <0.001 | 1,2-diacylglycerol-3-alpha-glucose alpha-1,2-galactosyltransferase | Teichoic acid biosynthesis |
| K13953 | 0.000532 | 0.000117 | <0.001 | alcohol dehydrogenase, propanol-preferring | Glycolysis / Gluconeogenesis, Fatty acid degradation, Tyrosine metabolism, Pyruvate metabolism, Chloroalkane and chloroalkene degradation, Naphthalene degradation, Retinol metabolism, Metabolism of xenobiotics by cytochrome P450, Drug metabolism - cytochrome P450, Metabolic pathways, Biosynthesis of secondary metabolites, Microbial metabolism in diverse environments, Degradation of aromatic compounds |
| K14153 | 0.000474 | 0.000118 | 0.003 | hydroxymethylpyrimidine kinase / phosphomethylpyrimidine kinase / thiamine-phosphate diphosphorylase | Thiamine metabolism, Metabolic pathways, Biosynthesis of cofactors |
| K14188 | 0.000612 | 0.000104 | <0.001 | D-alanine--poly(phosphoribitol) ligase subunit 2 | D-Amino acid metabolism, Teichoic acid biosynthesis, Metabolic pathways, Cationic antimicrobial peptide (CAMP) resistance, Two-component system, Staphylococcus aureus infection |
| K14205 | 0.000711 | 0.00012 | <0.001 | phosphatidylglycerol lysyltransferase | Cationic antimicrobial peptide (CAMP) resistance, Two-component system, Staphylococcus aureus infection |
| K14982 | 0.000538 | 0.000102 | <0.001 | two-component system, OmpR family, sensor histidine kinase CiaH | Two-component system, Quorum sensing |
| K15524 | 0.000603 | 0.000141 | 0.001 | 2-O-(6-phospho-alpha-D-mannosyl)-D-glycerate hydrolase |  |
| K16214 | 0.000541 | 9.92E-05 | <0.001 | UDP-N-acetylglucosamine kinase |  |
| **Other dairy products** | | |  |  |  |
| K01950 | -0.00006 | 0.000011 | <0.001 | NAD+ synthase (glutamine-hydrolysing) | Nicotinate and nicotinamide metabolism, Metabolic pathways, Biosynthesis of cofactors |
| **Vegetables** | |  |  |  |  |
| K00005 | -0.0008 | 0.000176 | 0.001 | glycerol dehydrogenase | Glycerolipid metabolism, Propanoate metabolism, Metabolic pathways |
| K00010 | -0.00066 | 0.000169 | 0.003 | myo-inositol 2-dehydrogenase / D-chiro-inositol 1-dehydrogenase | Streptomycin biosynthesis, Inositol phosphate metabolism, Metabolic pathways, Biosynthesis of secondary metabolites, Microbial metabolism in diverse environments |
| K00024 | 0.000138 | 3.43E-05 | 0.003 | malate dehydrogenase | Citrate cycle (TCA cycle), Cysteine and methionine metabolism, Pyruvate metabolism, Glyoxylate and dicarboxylate metabolism, Methane metabolism, Carbon fixation in photosynthetic organisms, Carbon fixation pathways in prokaryotes, Metabolic pathways, Biosynthesis of secondary metabolites, Microbial metabolism in diverse environments, Carbon metabolism |
| K00027 | -0.00058 | 0.000149 | 0.003 | malate dehydrogenase (oxaloacetate-decarboxylating) | Pyruvate metabolism, Carbon metabolism,  Two-component system |
| K00029 | 0.000129 | 3.28E-05 | 0.003 | malate dehydrogenase (oxaloacetate-decarboxylating)(NADP+) | Pyruvate metabolism, Carbon fixation in photosynthetic organisms, Metabolic pathways, Microbial metabolism in diverse environments, Carbon metabolism, PPAR signaling pathway |
| K00065 | -0.00072 | 0.000167 | 0.001 | 2-dehydro-3-deoxy-D-gluconate 5-dehydrogenase | Pentose and glucuronate interconversions,  Metabolic pathways |
| K00135 | -0.00062 | 0.000163 | 0.004 | succinate-semialdehyde dehydrogenase / glutarate-semialdehyde dehydrogenase | Alanine, aspartate and glutamate metabolism, Lysine degradation, Tyrosine metabolism, Butanoate metabolism, Nicotinate and nicotinamide metabolism, Metabolic pathways, Microbial metabolism in diverse environments |
| K00324 | -0.00086 | 0.000217 | 0.003 | proton-translocating NAD(P)+ transhydrogenase subunit alpha | Nicotinate and nicotinamide metabolism,  Metabolic pathways |
| K00325 | -0.00086 | 0.000212 | 0.002 | proton-translocating NAD(P)+ transhydrogenase subunit beta | Nicotinate and nicotinamide metabolism,  Metabolic pathways |
| K00366 | -0.00058 | 0.000157 | 0.005 | ferredoxin-nitrite reductase | Nitrogen metabolism, Metabolic pathways,  Microbial metabolism in diverse environments |
| K00549 | -0.00076 | 0.000191 | 0.003 | 5-methyltetrahydropteroyltriglutamate--homocysteine methyltransferase | Cysteine and methionine metabolism, Selenocompound metabolism, Metabolic pathways, Biosynthesis of secondary metabolites, Biosynthesis of amino acids |
| K00564 | -0.00066 | 0.000152 | 0.001 | 16S rRNA (guanine1207-N2)-methyltransferase |  |
| K00574 | -0.0008 | 0.000168 | 0.001 | cyclopropane-fatty-acyl-phospholipid synthase |  |
| K00598 | -0.00109 | 0.000221 | <0.001 | trans-aconitate 2-methyltransferase |  |
| K00639 | 0.000121 | 3.17E-05 | 0.004 | glycine C-acetyltransferase | Glycine, serine and threonine metabolism,  Metabolic pathways |
| K00681 | -0.00073 | 0.000178 | 0.002 | gamma-glutamyltranspeptidase / glutathione hydrolase | Taurine and hypotaurine metabolism,  Cyanoamino acid metabolism, Glutathione metabolism, Metabolic pathways |
| K00690 | -0.00086 | 0.000204 | 0.002 | sucrose phosphorylase | Starch and sucrose metabolism, Metabolic pathways |
| K00823 | -0.00073 | 0.000184 | 0.003 | 4-aminobutyrate aminotransferase | Alanine, aspartate and glutamate metabolism, beta-Alanine metabolism, Propanoate metabolism, Butanoate metabolism, Metabolic pathways, Microbial metabolism in diverse environments |
| K00851 | -0.0009 | 0.000217 | 0.002 | gluconokinase | Pentose phosphate pathway, Metabolic pathways, Biosynthesis of secondary metabolites, Microbial metabolism in diverse environments, Carbon metabolism |
| K00867 | -0.00069 | 0.000186 | 0.005 | type I pantothenate kinase | Pantothenate and CoA biosynthesis, Metabolic pathways, Biosynthesis of cofactors |
| K00872 | -0.00052 | 0.00013 | 0.003 | homoserine kinase | Glycine, serine and threonine metabolism, Metabolic pathways, Biosynthesis of secondary metabolites, Microbial metabolism in diverse environments, Biosynthesis of amino acids |
| K00880 | -0.00093 | 0.000185 | <0.001 | L-xylulokinase | Pentose and glucuronate interconversions, Ascorbate and aldarate metabolism, Metabolic pathways |
| K00881 | -0.00093 | 0.000211 | 0.001 | allose kinase | Fructose and mannose metabolism, Metabolic pathways, Microbial metabolism in diverse environments |
| K00945 | 7.45E-05 | 1.97E-05 | 0.004 | CMP/dCMP kinase | Pyrimidine metabolism, Metabolic pathways, Nucleotide metabolism |
| K00965 | -0.00059 | 0.000149 | 0.003 | UDPglucose--hexose-1-phosphate uridylyltransferase | Galactose metabolism, Amino sugar and nucleotide sugar metabolism, Metabolic pathways, Biosynthesis of nucleotide sugars, Prolactin signaling pathway |
| K00982 | -0.00083 | 0.000187 | 0.001 | [glutamine synthetase] adenylyltransferase / [glutamine synthetase]-adenylyl-L-tyrosine phosphorylase |  |
| K00989 | -0.00066 | 0.000154 | 0.001 | ribonuclease PH |  |
| K00990 | -0.00093 | 0.000216 | 0.001 | [protein-PII] uridylyltransferase | Two-component system |
| K00995 | -0.0006 | 0.000161 | 0.005 | CDP-diacylglycerol---glycerol-3-phosphate 3-phosphatidyltransferase | Glycerophospholipid metabolism, Metabolic pathways |
| K00997 | -0.00066 | 0.000165 | 0.003 | holo-[acyl-carrier protein] synthase | Pantothenate and CoA biosynthesis, Metabolic pathways |
| K01002 | -0.00064 | 0.000156 | 0.002 | phosphoglycerol transferase |  |
| K01011 | -0.00057 | 0.000153 | 0.005 | thiosulfate/3-mercaptopyruvate sulfurtransferase | Cysteine and methionine metabolism, Sulfur metabolism, Metabolic pathways, Microbial metabolism in diverse environments, Sulfur relay system |
| K01026 | -0.00061 | 0.000155 | 0.003 | propionate CoA-transferase | Pyruvate metabolism, Propanoate metabolism, Styrene degradation,Metabolic pathways, Microbial metabolism in diverse environments |
| K01159 | -0.00038 | 9.31E-05 | 0.002 | crossover junction endodeoxyribonuclease RuvC | Homologous recombination |
| K01193 | -0.00078 | 0.000195 | 0.003 | beta-fructofuranosidase | Galactose metabolism, Starch and sucrose metabolism, Metabolic pathways, Biosynthesis of secondary metabolites |
| K01223 | -0.00055 | 0.000135 | 0.003 | 6-phospho-beta-glucosidase | Glycolysis / Gluconeogenesis, Starch and sucrose metabolism |
| K01239 | -0.00078 | 0.000187 | 0.002 | purine nucleosidase | Purine metabolism, Metabolic pathways, Biosynthesis of secondary metabolites, Nucleotide metabolism |
| K01255 | -0.00066 | 0.00017 | 0.003 | leucyl aminopeptidase | Glutathione metabolism, Metabolic pathways |
| K01256 | -0.00052 | 0.000116 | 0.001 | aminopeptidase N | Glutathione metabolism, Metabolic pathways |
| K01271 | -0.00067 | 0.000169 | 0.003 | Xaa-Pro dipeptidase |  |
| K01304 | -0.00065 | 0.000167 | 0.003 | pyroglutamyl-peptidase |  |
| K01354 | -0.00093 | 0.0002 | 0.001 | oligopeptidase B | Chagas disease, African trypanosomiasis |
| K01356 | -0.00057 | 0.000143 | 0.003 | repressor LexA |  |
| K01419 | -0.00075 | 0.00018 | 0.002 | ATP-dependent HslUV protease, peptidase subunit HslV |  |
| K01439 | -0.00066 | 0.000148 | 0.001 | succinyl-diaminopimelate desuccinylase | Lysine biosynthesis, Metabolic pathways,  Microbial metabolism in diverse environments, Biosynthesis of amino acids |
| K01485 | -0.00072 | 0.000187 | 0.004 | cytosine/creatinine deaminase | Pyrimidine metabolism, Arginine and proline metabolism, Metabolic pathways, Nucleotide metabolism |
| K01487 | -0.0007 | 0.000185 | 0.004 | guanine deaminase | Purine metabolism, Metabolic pathways,  Nucleotide metabolism |
| K01494 | -0.00053 | 0.000143 | 0.005 | dCTP deaminase | Pyrimidine metabolism, Metabolic pathways, Nucleotide metabolism |
| K01507 | -0.00055 | 0.000142 | 0.004 | inorganic pyrophosphatase | Oxidative phosphorylation |
| K01523 | -0.00064 | 0.000171 | 0.004 | phosphoribosyl-ATP pyrophosphohydrolase | Histidine metabolism, Metabolic pathways,  Biosynthesis of secondary metabolites,  Biosynthesis of amino acids |
| K01575 | -0.00085 | 0.000187 | 0.001 | acetolactate decarboxylase | Butanoate metabolism, C5-Branched dibasic acid metabolism, Biosynthesis of secondary metabolites, 2-Oxocarboxylic acid metabolism |
| K01579 | 0.000101 | 2.40E-05 | 0.002 | aspartate 1-decarboxylase | beta-Alanine metabolism, Pantothenate and CoA biosynthesis, Metabolic pathways, Biosynthesis of secondary metabolites, Biosynthesis of cofactors |
| K01581 | -0.00087 | 0.000218 | 0.003 | ornithine decarboxylase | Arginine and proline metabolism, Glutathione metabolism, Metabolic pathways, Biosynthesis of secondary metabolites, Efferocytosis |
| K01595 | -0.0008 | 0.000181 | 0.001 | phosphoenolpyruvate carboxylase | Pyruvate metabolism, Methane metabolism, Carbon fixation in photosynthetic organisms, Carbon fixation pathways in prokaryotes, Metabolic pathways, Microbial metabolism in diverse environments, Carbon metabolism |
| K01607 | -0.00049 | 0.000126 | 0.003 | 4-carboxymuconolactone decarboxylase | Benzoate degradation, Metabolic pathways,  Microbial metabolism in diverse environments, Degradation of aromatic compounds |
| K01626 | -0.00063 | 0.00016 | 0.003 | 3-deoxy-7-phosphoheptulonate synthase | Phenylalanine, tyrosine and tryptophan biosynthesis, Metabolic pathways, Biosynthesis of secondary metabolites,  Biosynthesis of amino acids, Quorum sensing |
| K01664 | -0.00083 | 0.000184 | 0.001 | para-aminobenzoate synthetase component II | Folate biosynthesis, Biosynthesis of cofactors |
| K01673 | -0.00059 | 0.00013 | 0.001 | carbonic anhydrase | Nitrogen metabolism, Metabolic pathways |
| K01681 | -0.00048 | 0.000119 | 0.003 | aconitate hydratase | Citrate cycle (TCA cycle), Glyoxylate and dicarboxylate metabolism, Carbon fixation pathways in prokaryotes, Metabolic pathways, Biosynthesis of secondary metabolites, Microbial metabolism in diverse environments, Carbon metabolism, 2-Oxocarboxylic acid metabolism, Biosynthesis of amino acids |
| K01739 | -0.00073 | 0.000166 | 0.001 | cystathionine gamma-synthase | Cysteine and methionine metabolism, Selenocompound metabolism, Sulfur metabolism, Metabolic pathways, Biosynthesis of secondary metabolites, Biosynthesis of amino acids |
| K01753 | -0.0007 | 0.000189 | 0.005 | D-serine dehydratase | Glycine, serine and threonine metabolism, D-Amino acid metabolism, Metabolic pathways |
| K01760 | -0.00081 | 0.000191 | 0.002 | cysteine-S-conjugate beta-lyase | Cysteine and methionine metabolism, Selenocompound metabolism, Metabolic pathways, Biosynthesis of secondary metabolites, Biosynthesis of amino acids |
| K01779 | -0.00079 | 0.000201 | 0.003 | aspartate racemase | Alanine, aspartate and glutamate metabolism, D-Amino acid metabolism, Nonribosomal peptide structures, Metabolic pathways |
| K01787 | 0.000171 | 4.54E-05 | 0.004 | N-acylglucosamine 2-epimerase | Amino sugar and nucleotide sugar metabolism, Metabolic pathways |
| K01788 | -0.0006 | 0.000131 | 0.001 | N-acylglucosamine-6-phosphate 2-epimerase | Amino sugar and nucleotide sugar metabolism, Metabolic pathways |
| K01807 | -0.00059 | 0.000139 | 0.002 | ribose 5-phosphate isomerase A | Pentose phosphate pathway, Carbon fixation in photosynthetic organisms, Metabolic pathways, Biosynthesis of secondary metabolites, Microbial metabolism in diverse environments, Carbon metabolism, Biosynthesis of amino acids |
| K01847 | 0.000116 | 3.13E-05 | 0.005 | methylmalonyl-CoA mutase | Valine, leucine and isoleucine degradation, Glyoxylate and dicarboxylate metabolism,  Propanoate metabolism, Carbon fixation pathways in prokaryotes, Metabolic pathways, Microbial metabolism in diverse environments, Carbon metabolism |
| K01878 | -0.00063 | 0.000162 | 0.003 | glycyl-tRNA synthetase alpha chain | Aminoacyl-tRNA biosynthesis |
| K01879 | -0.00063 | 0.000156 | 0.003 | glycyl-tRNA synthetase beta chain | Aminoacyl-tRNA biosynthesis |
| K01886 | 9.23E-05 | 2.45E-05 | 0.004 | glutaminyl-tRNA synthetase | Aminoacyl-tRNA biosynthesis, Metabolic pathways |
| K01902 | -0.00053 | 0.000137 | 0.004 | succinyl-CoA synthetase alpha subunit | Citrate cycle (TCA cycle), Propanoate metabolism, C5-Branched dibasic acid metabolism, Carbon fixation pathways in prokaryotes, Metabolic pathways, Biosynthesis of secondary metabolites, Microbial metabolism in diverse environments, Carbon metabolism |
| K01903 | -0.00053 | 0.000138 | 0.004 | succinyl-CoA synthetase beta subunit | Citrate cycle (TCA cycle), Propanoate metabolism, C5-Branched dibasic acid metabolism, Carbon fixation pathways in prokaryotes, Metabolic pathways,  Biosynthesis of secondary metabolites, Microbial metabolism in diverse environments, Carbon metabolism |
| K01918 | 8.17E-05 | 2.13E-05 | 0.004 | pantoate--beta-alanine ligase | beta-Alanine metabolism, Pantothenate and CoA biosynthesis, Metabolic pathways, Biosynthesis of secondary metabolites, Biosynthesis of cofactors |
| K01919 | -0.00073 | 0.000148 | <0.001 | glutamate--cysteine ligase | Cysteine and methionine metabolism, Glutathione metabolism, Metabolic pathways, Biosynthesis of cofactors |
| K01962 | -0.00056 | 0.000145 | 0.004 | acetyl-CoA carboxylase carboxyl transferase subunit alpha | Fatty acid biosynthesis, Pyruvate metabolism, Propanoate metabolism, Carbon fixation pathways in prokaryotes, Metabolic pathways, Biosynthesis of secondary metabolites, Microbial metabolism in diverse environments, Carbon metabolism, Fatty acid metabolism |
| K02017 | -0.00054 | 0.000135 | 0.003 | molybdate transport system ATP-binding protein | ABC transporters |
| K02045 | -0.00075 | 0.000189 | 0.003 | sulfate/thiosulfate transport system ATP-binding protein | Sulfur metabolism, ABC transporters |
| K02083 | -0.00079 | 0.000211 | 0.004 | allantoate deiminase | Purine metabolism, Metabolic pathways, Microbial metabolism in diverse environments |
| K02446 | -0.0007 | 0.000185 | 0.004 | fructose-1,6-bisphosphatase II | Glycolysis / Gluconeogenesis, Pentose phosphate pathway, Fructose and mannose metabolism, Methane metabolism, Carbon fixation in photosynthetic organisms, Metabolic pathways, Biosynthesis of secondary metabolites, Microbial metabolism in diverse environments, Carbon metabolism |
| K02564 | 0.00011 | 2.65E-05 | 0.002 | glucosamine-6-phosphate deaminase | Amino sugar and nucleotide sugar metabolism, Metabolic pathways |
| K02744 | -0.00059 | 0.000134 | 0.001 | N-acetylgalactosamine PTS system EIIA component | Galactose metabolism, Metabolic pathways,  Phosphotransferase system (PTS) |
| K02777 | -0.00074 | 0.00019 | 0.003 | sugar PTS system EIIA component | Glycolysis / Gluconeogenesis, Starch and sucrose metabolism, Amino sugar and nucleotide sugar metabolism, Metabolic pathways, Biofilm formation - Escherichia coli, Phosphotransferase system (PTS), Biofilm formation - Vibrio cholerae |
| K02781 | -0.00076 | 0.000198 | 0.004 | glucitol/sorbitol PTS system EIIA component | Fructose and mannose metabolism, Metabolic pathways, Phosphotransferase system (PTS) |
| K02794 | -0.00057 | 0.00014 | 0.002 | mannose PTS system EIIAB component | Fructose and mannose metabolism, Amino sugar and nucleotide sugar metabolism, Metabolic pathways, Phosphotransferase system (PTS) |
| K02798 | -0.00075 | 0.000192 | 0.003 | mannitol PTS system EIIA component | Fructose and mannose metabolism, Metabolic pathways, Phosphotransferase system (PTS) |
| K02821 | -0.0008 | 0.000183 | 0.001 | ascorbate PTS system EIIA or EIIAB component | Ascorbate and aldarate metabolism, Metabolic pathways, Microbial metabolism in diverse environments, Phosphotransferase system (PTS) |
| K02822 | -0.00075 | 0.000165 | 0.001 | ascorbate PTS system EIIB component | Ascorbate and aldarate metabolism, Metabolic pathways, Microbial metabolism in diverse environments, Phosphotransferase system (PTS) |
| K02851 | -0.00087 | 0.000184 | 0.001 | UDP-GlcNAc:undecaprenyl-phosphate/decaprenyl-phosphate GlcNAc-1-phosphate transferase | O-Antigen repeat unit biosynthesis, Exopolysaccharide biosynthesis, Teichoic acid biosynthesis, Arabinogalactan biosynthesis – Mycobacterium, Metabolic pathways |
| K03079 | -0.001 | 0.0002 | <0.001 | L-ribulose-5-phosphate 3-epimerase | Pentose and glucuronate interconversions, Ascorbate and aldarate metabolism, Metabolic pathways, Microbial metabolism in diverse environments |
| K03148 | -0.00085 | 0.000203 | 0.002 | sulfur carrier protein ThiS adenylyltransferase | Thiamine metabolism, Metabolic pathways, Biosynthesis of cofactors, Sulfur relay system |
| K03183 | 0.000101 | 2.69E-05 | 0.004 | demethylmenaquinone methyltransferase / 2-methoxy-6-polyprenyl-1,4-benzoquinol methylase | Ubiquinone and other terpenoid-quinone biosynthesis, Metabolic pathways, Biosynthesis of secondary metabolites, Biosynthesis of cofactors |
| K03216 | -0.00061 | 0.000157 | 0.003 | tRNA (cytidine/uridine-2'-O-)-methyltransferase |  |
| K03431 | -0.00069 | 0.00016 | 0.001 | phosphoglucosamine mutase | Amino sugar and nucleotide sugar metabolism, Metabolic pathways, Biosynthesis of nucleotide sugars |
| K03474 | 0.000131 | 3.34E-05 | 0.003 | pyridoxine 5-phosphate synthase | Vitamin B6 metabolism, Metabolic pathways, Biosynthesis of cofactors |
| K03578 | -0.00087 | 0.000217 | 0.003 | ATP-dependent helicase HrpA |  |
| K03684 | -0.0007 | 0.000184 | 0.004 | ribonuclease D |  |
| K03767 | -0.00087 | 0.000197 | 0.001 | peptidyl-prolyl cis-trans isomerase A (cyclophilin A) | Cationic antimicrobial peptide (CAMP) resistance, Viral life cycle - HIV-1, Necroptosis |
| K03784 | -0.0006 | 0.000146 | 0.002 | purine-nucleoside phosphorylase | Purine metabolism, Nicotinate and nicotinamide metabolism, Metabolic pathways, Biosynthesis of secondary metabolites, Nucleotide metabolism |
| K03789 | -0.00056 | 0.000148 | 0.004 | [ribosomal protein S18]-alanine N-acetyltransferase |  |
| K03800 | -0.00054 | 0.000132 | 0.003 | lipoate---protein ligase | Lipoic acid metabolism, Metabolic pathways, Biosynthesis of cofactors |
| K03830 | -0.00061 | 0.000146 | 0.002 | putative acetyltransferase |  |
| K04042 | -0.00054 | 0.000141 | 0.003 | bifunctional UDP-N-acetylglucosamine pyrophosphorylase / glucosamine-1-phosphate N-acetyltransferase | Amino sugar and nucleotide sugar metabolism, O-Antigen nucleotide sugar biosynthesis, Metabolic pathways, Biosynthesis of nucleotide sugars |
| K04072 | -0.00076 | 0.000176 | 0.002 | acetaldehyde dehydrogenase / alcohol dehydrogenase | Glycolysis / Gluconeogenesis, Fatty acid degradation, Tyrosine metabolism, Pyruvate metabolism, Chloroalkane and chloroalkene degradation, Naphthalene degradation, Butanoate metabolism, Metabolic pathways, Biosynthesis of secondary metabolites, Microbial metabolism in diverse environments, Degradation of aromatic compounds, Amoebiasis |
| K04771 | -0.0006 | 0.000152 | 0.003 | serine protease Do | Cationic antimicrobial peptide (CAMP) resistance, Two-component system |
| K05341 | -0.00079 | 0.000171 | 0.001 | amylosucrase | Starch and sucrose metabolism, Metabolic pathways |
| K05396 | -0.00069 | 0.000172 | 0.003 | D-cysteine desulfhydrase | Cysteine and methionine metabolism, D-Amino acid metabolism, Metabolic pathways |
| K05878 | -0.00067 | 0.000155 | 0.001 | phosphoenolpyruvate---glycerone phosphotransferase subunit DhaK | Glycerolipid metabolism, Metabolic pathways |
| K05879 | -0.00055 | 0.000146 | 0.004 | phosphoenolpyruvate---glycerone phosphotransferase subunit DhaL | Glycerolipid metabolism, Metabolic pathways |
| K06269 | 0.000324 | 8.52E-05 | 0.004 | serine/threonine-protein phosphatase PP1 catalytic subunit | mRNA surveillance pathway, cGMP-PKG signaling pathway, cAMP signaling pathway, Meiosis – yeast, Oocyte meiosis, Cellular senescence, Adrenergic signaling in cardiomyocytes, Vascular smooth muscle contraction, Hippo signaling pathway, Focal adhesion, Platelet activation, Long-term potentiation, Dopaminergic synapse, Inflammatory mediator regulation of TRP channels, Regulation of actin cytoskeleton, Insulin signaling pathway, Oxytocin signaling pathway, Insulin resistance, Amphetamine addiction, Alcoholism, Herpes simplex virus 1 infection, Proteoglycans in cancer, Diabetic cardiomyopathy |
| K06859 | -0.0005 | 0.000128 | 0.003 | glucose-6-phosphate isomerase, archaeal | Glycolysis / Gluconeogenesis, Pentose phosphate pathway, Starch and sucrose metabolism, Amino sugar and nucleotide sugar metabolism, Metabolic pathways, Biosynthesis of secondary metabolites, Microbial metabolism in diverse environments, Carbon metabolism |
| K07107 | 0.000113 | 2.79E-05 | 0.003 | acyl-CoA thioester hydrolase |  |
| K07141 | -0.00058 | 0.000153 | 0.004 | molybdenum cofactor cytidylyltransferase | Folate biosynthesis, Metabolic pathways |
| K07246 | -0.00078 | 0.000201 | 0.003 | tartrate dehydrogenase/decarboxylase / D-malate dehydrogenase | Glyoxylate and dicarboxylate metabolism, Butanoate metabolism, Metabolic pathways |
| K07250 | -0.00092 | 0.000192 | 0.001 | 4-aminobutyrate aminotransferase / (S)-3-amino-2-methylpropionate transaminase / 5-aminovalerate transaminase | Alanine, aspartate and glutamate metabolism, Valine, leucine and isoleucine degradation, Lysine degradation, beta-Alanine metabolism, Propanoate metabolism, Butanoate metabolism, Metabolic pathways, Microbial metabolism in diverse environments |
| K07258 | -0.00062 | 0.000153 | 0.003 | serine-type D-Ala-D-Ala carboxypeptidase (penicillin-binding protein 5/6) | Peptidoglycan biosynthesis, Metabolic pathways |
| K07260 | -0.00058 | 0.000137 | 0.002 | zinc D-Ala-D-Ala carboxypeptidase | Peptidoglycan biosynthesis, Metabolic pathways, Vancomycin resistance, Two-component system |
| K07272 | -0.00086 | 0.000227 | 0.004 | rhamnosyltransferase |  |
| K07317 | -0.00086 | 0.000219 | 0.003 | adenine-specific DNA-methyltransferase |  |
| K07588 | 0.000123 | 3.22E-05 | 0.004 | Methylmalonic aciduria |  |
| K07642 | -0.00079 | 0.000207 | 0.004 | two-component system, OmpR family, sensor histidine kinase BaeS | Two-component system |
| K07749 | -0.00071 | 0.00019 | 0.004 | formyl-CoA transferase |  |
| K08256 | -0.00064 | 0.000157 | 0.002 | phosphatidyl-myo-inositol alpha-mannosyltransferase | Lipoarabinomannan (LAM) biosynthesis, Metabolic pathways |
| K08281 | -0.00093 | 0.000185 | <0.001 | nicotinamidase/pyrazinamidase | Nicotinate and nicotinamide metabolism, Metabolic pathways, Biosynthesis of cofactors |
| K08296 | -0.00082 | 0.000174 | 0.001 | phosphohistidine phosphatase |  |
| K08300 | -0.00082 | 0.000185 | 0.001 | ribonuclease E | RNA degradation |
| K08316 | -0.00071 | 0.000141 | <0.001 | 16S rRNA (guanine966-N2)-methyltransferase |  |
| K08678 | 0.000192 | 4.95E-05 | 0.003 | UDP-glucuronate decarboxylase | Amino sugar and nucleotide sugar metabolism, Metabolic pathways, Biosynthesis of nucleotide sugars |
| K09001 | -0.0007 | 0.00017 | 0.002 | anhydro-N-acetylmuramic acid kinase | Amino sugar and nucleotide sugar metabolism, Metabolic pathways, Biosynthesis of nucleotide sugars |
| K09994 | -0.00066 | 0.000156 | 0.002 | (aminoalkyl)phosphonate N-acetyltransferase | Phosphonate and phosphinate metabolism |
| K10213 | -0.0007 | 0.000166 | 0.002 | ribosylpyrimidine nucleosidase | Pyrimidine metabolism, Metabolic pathways,  Nucleotide metabolism |
| K10708 | -0.00054 | 0.000132 | 0.002 | fructoselysine 6-phosphate deglycase |  |
| K10805 | -0.00089 | 0.000217 | 0.002 | acyl-CoA thioesterase II | Biosynthesis of unsaturated fatty acids |
| K12527 | -0.00063 | 0.000171 | 0.005 | putative selenate reductase | Selenocompound metabolism |
| K12700 | -0.00081 | 0.000189 | 0.001 | non-specific riboncleoside hydrolase | Pyrimidine metabolism, Metabolic pathways, Nucleotide metabolism |
| K13788 | -0.00088 | 0.000215 | 0.002 | phosphate acetyltransferase | Taurine and hypotaurine metabolism, Pyruvate metabolism, Propanoate metabolism, Methane metabolism, Carbon fixation pathways in prokaryotes, Metabolic pathways, Microbial metabolism in diverse environments, Carbon metabolism |
| K13829 | -0.00111 | 0.000228 | <0.001 | shikimate kinase / 3-dehydroquinate synthase | Phenylalanine, tyrosine and tryptophan biosynthesis, Metabolic pathways, Biosynthesis of secondary metabolites, Biosynthesis of amino acids |
| K13953 | -0.00065 | 0.000174 | 0.004 | alcohol dehydrogenase, propanol-preferring | Glycolysis / Gluconeogenesis, Fatty acid degradation, Tyrosine metabolism, Pyruvate metabolism, Chloroalkane and chloroalkene degradation, Naphthalene degradation, Retinol metabolism, Metabolism of xenobiotics by cytochrome P450, Drug metabolism - cytochrome P450, Metabolic pathways, Biosynthesis of secondary metabolites, Microbial metabolism in diverse environments, Degradation of aromatic compounds |
| K14260 | -0.00084 | 0.000167 | <0.001 | alanine-synthesizing transaminase | Arginine biosynthesis, Alanine, aspartate and glutamate metabolism, Valine, leucine and isoleucine biosynthesis, Metabolic pathways, Biosynthesis of secondary metabolites, 2-Oxocarboxylic acid metabolism, Biosynthesis of amino acids |
| K16148 | -0.00091 | 0.000228 | 0.003 | alpha-maltose-1-phosphate synthase | Starch and sucrose metabolism, Metabolic pathways |
| **Cereals** |  |  |  |  |  |
| K00324 | 0.001371 | 0.000316 | 0.002 | proton-translocating NAD(P)+ transhydrogenase subunit alpha | Nicotinate and nicotinamide metabolism, Metabolic pathways |
| K00325 | 0.001286 | 0.000308 | 0.004 | proton-translocating NAD(P)+ transhydrogenase subunit beta | Nicotinate and nicotinamide metabolism, Metabolic pathways |
| K00690 | 0.001227 | 0.000298 | 0.004 | sucrose phosphorylase | Starch and sucrose metabolism, Metabolic pathways |
| K00886 | 0.001653 | 0.000339 | 0.001 | polyphosphate glucokinase | Glycolysis / Gluconeogenesis, Amino sugar and nucleotide sugar metabolism, Metabolic pathways, Biosynthesis of secondary metabolites, Microbial metabolism in diverse environments, Carbon metabolism, Biosynthesis of nucleotide sugars |
| K01198 | 0.001559 | 0.000349 | 0.001 | xylan 1,4-beta-xylosidase | Amino sugar and nucleotide sugar metabolism, Metabolic pathways |
| K01210 | 0.001928 | 0.000415 | 0.001 | glucan 1,3-beta-glucosidase | Starch and sucrose metabolism, Metabolic pathways |
| K01259 | 0.001468 | 0.000327 | 0.001 | proline iminopeptidase | Arginine and proline metabolism, Metabolic pathways |
| K01436 | 0.001596 | 0.000348 | 0.001 | amidohydrolase |  |
| K01621 | 0.001452 | 0.000317 | 0.001 | xylulose-5-phosphate/fructose-6-phosphate phosphoketolase | Pentose phosphate pathway, Microbial metabolism in diverse environments |
| K01697 | 0.001602 | 0.000343 | 0.001 | cystathionine beta-synthase | Glycine, serine and threonine metabolism, Cysteine and methionine metabolism, Metabolic pathways, Biosynthesis of secondary metabolites, Biosynthesis of amino acids |
| K01779 | 0.00123 | 0.000293 | 0.004 | aspartate racemase | Alanine, aspartate and glutamate metabolism, D-Amino acid metabolism, Nonribosomal peptide structures, Metabolic pathways |
| K03148 | 0.001234 | 0.000296 | 0.004 | sulfur carrier protein ThiS adenylyltransferase | Thiamine metabolism, Metabolic pathways, Biosynthesis of cofactors, Sulfur relay system |
| K03578 | 0.001351 | 0.000315 | 0.003 | ATP-dependent helicase HrpA |  |
| K03707 | 0.001461 | 0.000334 | 0.002 | thiaminase (transcriptional activator TenA) | Thiamine metabolism, Metabolic pathways Biosynthesis of cofactors |
| K03929 | 0.001522 | 0.000333 | 0.001 | para-nitrobenzyl esterase |  |
| K04092 | 0.0018 | 0.000379 | 0.001 | chorismate mutase | Phenylalanine, tyrosine and tryptophan biosynthesis, Metabolic pathways, Biosynthesis of secondary metabolites, Biosynthesis of amino acids |
| K05351 | 0.001941 | 0.000382 | 0.001 | D-xylulose reductase | Pentose and glucuronate interconversions, Metabolic pathways |
| K05364 | 0.001267 | 0.000293 | 0.003 | penicillin-binding protein A | Peptidoglycan biosynthesis, Metabolic pathways |
| K07272 | 0.001414 | 0.000331 | 0.003 | rhamnosyltransferase |  |
| K07442 | 0.001577 | 0.000347 | 0.001 | tRNA (adenine57-N1/adenine58-N1)-methyltransferase catalytic subunit |  |
| K07768 | 0.001744 | 0.000368 | 0.001 | two-component system, OmpR family, sensor histidine kinase SenX3 | Two-component system |
| K10805 | 0.001339 | 0.000316 | 0.003 | acyl-CoA thioesterase II | Biosynthesis of unsaturated fatty acids |
| K11263 | 0.001569 | 0.000339 | 0.001 | acetyl-CoA/propionyl-CoA carboxylase, biotin carboxylase, biotin carboxyl carrier protein | Fatty acid biosynthesis, Valine, leucine and isoleucine degradation, Pyruvate metabolism, Glyoxylate and dicarboxylate metabolism, Propanoate metabolism, Metabolic pathways, Biosynthesis of secondary metabolites, Microbial metabolism in diverse environments, Carbon metabolism, Fatty acid metabolism |
| K11533 | 0.001799 | 0.000374 | 0.001 | fatty acid synthase, bacteria type | Fatty acid biosynthesis, Metabolic pathways, Fatty acid metabolism, Insulin resistance |
| K13571 | 0.001715 | 0.000367 | 0.001 | proteasome accessory factor A |  |
| K13788 | 0.001308 | 0.000313 | 0.004 | phosphate acetyltransferase | Taurine and hypotaurine metabolism, Pyruvate metabolism, Propanoate metabolism, Methane metabolism, Carbon fixation pathways in prokaryotes, Metabolic pathways, Microbial metabolism in diverse environments, Carbon metabolism |
| K13940 | 0.001388 | 0.000303 | 0.001 | dihydroneopterin aldolase / 2-amino-4-hydroxy-6-hydroxymethyldihydropteridine diphosphokinase | Folate biosynthesis, Metabolic pathways, Biosynthesis of cofactors |
| K16147 | 0.001664 | 0.000354 | 0.001 | starch synthase (maltosyl-transferring) | Starch and sucrose metabolism, Metabolic pathways |
| BH; Benjamini-Hocberg  The models were adjusted for age, sex, BMI, smoking status, usage of possible microbiome altering medications (metformin and psycholeptics/psychoanaleptics) and total energy intake. | | | | | |
